# The costs and benefits of symbiotic interactions: variable effects of rhizobia and arbuscular mycorrhizae on *Vigna radiata* accessions

**DOI:** 10.1186/s12870-024-05488-5

**Published:** 2024-08-15

**Authors:** Chih-Cheng Chien, Shang-Ying Tien, Shu-Yi Yang, Cheng-Ruei Lee

**Affiliations:** 1 Institute of Ecology and Evolutionary Biology, National Taiwan University, Taipei, Taiwan; 2 Institute of Plant Biology, National Taiwan University, Taipei, Taiwan

**Keywords:** Mungbean accessions, Rhizobia, Arbuscular mycorrhizal fungus, Symbiosis, Synergetic effects, *Vigna radiata*, Mutualism, Multiple mutualist effects, Heritability, Fitness

## Abstract

**Background:**

The symbiosis among plants, rhizobia, and arbuscular mycorrhizal fungi (AMF) is one of the most well-known symbiotic relationships in nature. However, it is still unclear how bilateral/tripartite symbiosis works under resource-limited conditions and the diverse genetic backgrounds of the host.

**Results:**

Using a full factorial design, we manipulated mungbean accessions/subspecies, rhizobia, and AMF to test their effects on each other. Rhizobia functions as a typical facilitator by increasing plant nitrogen content, plant weight, chlorophyll content, and AMF colonization. In contrast, AMF resulted in a tradeoff in plants (reducing biomass for phosphorus acquisition) and behaved as a competitor in reducing rhizobia fitness (nodule weight). Plant genotype did not have a significant effect on AMF fitness, but different mungbean accessions had distinct rhizobia affinities. In contrast to previous studies, the positive relationship between plant and rhizobia fitness was attenuated in the presence of AMF, with wild mungbean being more responsive to the beneficial effect of rhizobia and attenuation by AMF.

**Conclusions:**

We showed that this complex tripartite relationship does not unconditionally benefit all parties. Moreover, rhizobia species and host genetic background affect the symbiotic relationship significantly. This study provides a new opportunity to re-evaluate the relationships between legume plants and their symbiotic partners.

**Supplementary Information:**

The online version contains supplementary material available at 10.1186/s12870-024-05488-5.

## Background

Since the emergence of life on Earth approximately 3.5 billion years ago, organisms have undergone a continuous process of evolution and adaptation in response to changing environmental conditions. This process has led to the development of intricate relationships among species, including mutualistic or symbiotic relationships, where organisms cooperate to enhance their survival and reproductive success [1]. Symbiosis, a fundamental aspect of ecological interactions, involves close and often interdependent relationships between different species. However, despite its importance, symbiosis is a fragile system susceptible to disruption when environmental conditions fluctuate. An example of this fragility is the relationship between corals and zooxanthellae (*Symbiodinium* spp.), where increases in temperature can lead to the expulsion of zooxanthellae by corals, resulting in the breakdown of the symbiotic partnership [2].

Moreover, along the continuum from mutualism to parasitism, where symbiotic relationships can shift in nature, various factors contribute to this dynamic process. The transition from mutualism to parasitism occurs when the costs associated with symbiosis surpass the benefits, leading to a change in the nature of the interaction [3, 4]. This phenomenon is influenced by numerous ecological and evolutionary factors, such as changes in environmental conditions, resource availability, and genetic adaptations within the interacting species.

Furthermore, symbiotic relationships are not limited to only two organisms. Additional partners can become involved, resulting in what is termed multiple mutualistic effects (MMEs) [5]. The inclusion of multiple partners in a symbiotic network adds layers of complexity to interactions, potentially altering the dynamics of mutualism and introducing new challenges for the participating organisms.

In such complex networks, a focal species often interacts with several other organisms, playing a central role in mediating resource allocation and maintaining balance within the symbiotic system [5]. This mediation may involve the distribution of nutrients, energy flow, or other essential resources necessary for the sustenance of all partners involved. As a result, the interplay between multiple organisms within a symbiotic network can significantly influence the stability and evolution of mutualistic relationships, shaping the overall structure and function of ecosystems.

In plants, the most well-known example of a multipartner symbiosis system is the legume-rhizobia-AMF system. Rhizobia are N-fixing bacteria that fix atmospheric nitrogen (N_2_) and transform it into inorganic N by forming nodules, providing an essential N source to the host. In exchange, rhizobia acquire carbohydrates from the host and uses them as energy for not only nitrogen fixation but also its growth and maintenance. On the other hand, AMF form symbiotic partnerships with 70–90% of land plants, aiding in the extraction of phosphorus (P) from the soil [6]. In exchange, the host plant contributes approximately 10%-23% of photosynthesis products to AMF [7]. Previous studies have investigated the effects of dual inoculation. For example, dual inoculation improved the growth of *Medicago truncatula* and *V. radiata* but not of *Vicia faba* [8–10]. Although it is known that environmental factors and microbial strains may affect symbiosis [4, 11, 12], recent studies have not discussed in depth the effects of plant genotype on bilateral/tripartite symbiosis under adverse conditions, and studies investigating the mutual effects of these factors on each other are rare. In addition, while the partners might benefit each other under standard conditions, it is unclear whether or how their relationship would break under a limited resource supply.

Here, we focused on the legume *V. radiata*. It is one of the most important legume crops in Asia and serves as green manure during the intercropping period. A previous study investigated the genetic structure and phenotypic properties of pan-Asian mungbean cultivars [13]. Briefly, cultivated mungbean can be divided into four geographically and genetically structured groups: South Asia, Southeast Asia, East Asia, and Central Asia, each with distinct morphology, yield components, and drought tolerance abilities [13]. In addition, wild progenitors are distributed across Australia and southern Asia [14]. Mungbean is, therefore, a valuable target for studying the symbiosis among hosts and microbes under different host genetic backgrounds.

To understand the symbiosis among rhizobia, AMF, and mungbean, we chose two rhizobia belonging to slow-growing *Bradyrhizobium* and fast-growing *Sinorhizobium* genera, owing to their differences in sucrose utilization and acid production [15, 16], in the symbiotic system. These two genera are the dominant rhizobia in the mungbean-rhizobia interaction system in different areas [17–22]. Additionally, we used *Rhizophagus irregularis* (*Ri*) in this study since it is a widely distributed model AMF worldwide, and its genome has been sequenced [23–26]. In addition, a previous study has reported that *Ri* could increase the yield of mungbean [27].

Previous studies have shown that dual inoculation of rhizobia and AMF could enhance the growth or seed production of mungbean [9, 28–30]. However, the symbiotic outcome depends considerably on the compatibility of the plant and AMF [31]. These studies focus on the relationship among AMF, *Bj* (rhizobia), and mungbean, but only one mungbean accession is used. It remains unknown whether mungbean genetic background affects the symbiotic relationship and, more importantly, whether or how wild or cultivated mungbean differ in symbiotic efficacy. Particularly, we wondered 1) if the dual inoculation of both *Ri* and *Bj* is better than single inoculation for the host, and 2) if the symbiotic outcome would be different using different host accessions under nutrient-limited conditions.

While *Bradyrhizobium* and *Sinorhizobium* are two main rhizobia that interacted with mungbean [17–22], most in-depth studies focused only on the mungbean-*Bradyrhizobium* interaction [9, 28, 32]. Given that there are few reports on the symbiotic efficiency of these two genera on various genetic backgrounds of mungbean, it would be valuable to investigate the mungbean genotype-rhizobia species interaction.

In this study, we used different accessions of *V. radiata* to investigate their bilateral/tripartite symbiosis with *Ri* and/or *Bj.* In addition, we performed symbiosis under N- and P-limited conditions to evaluate the nutrient uptake ability of the microbes under symbiosis. Using the three-way treatment manipulating the three partners in this symbiosis relationship, we are able to determine the effects of *Ri* and *Bj* on the plant traits as well as the rhizobia- and AMF-related traits under nutrient-limited conditions. Moreover, this study also provides additional results for the rhizobia-mungbean combinations with the highest affinity, contributing to future agronomic applications.

## Methods

### Plant material and growth conditions

Twenty accessions of *V. radiata* used in this study were obtained from the Australian Grains Genebank and the World Vegetable Center, including five *V. radiata* spp. *sublobata* (Accession: BCP_075, CPI106939, CQ2242, CQ3649, Karumbyar) and fifteen *V. radiata* spp. *radiata* (Fig. S1A and Table S1)*.* According to population genetics analysis [13], *V. radiata* could be separated into four genetic groups: East Asian, Central Asian, Southeast Asian, and South Asian. We therefore chose at least one accession to represent each group.

For the tripartite symbiosis experiments (Exp. 1 hereafter), a total of ten accessions, including those before (spp. *sublobata*, five accessions) and after (spp. *radiata*, five accessions) domestication, exhibited genetic variation in *V. radiata* worldwide. For the rhizobia efficiency experiments (Exp. 2 hereafter), ten additional cultivars were selected to test the interaction effect between the two rhizobia species and mungbean. In the assays, the East Asian and Central Asian mungbean groups were combined into one group, the North Asian group. Since *V. radiata* is a predominantly self-pollinating plant and the accessions were inbred in the laboratory for a few generations, the seeds of the same accession were used as replicates in all experiments.

All seeds were surface-sterilized with 1% sodium hypochlorite solution (1% NaOCl) for 30 min and washed three times with dH_2_O. Surface-disinfected mungbean seeds were submerged in dH_2_O in 15 cm Petri dishes and left in the dark for 3 days. The dH_2_O was renewed every day. After germination, the seedlings were transferred to 7 × 7 cm square pots with 250 ml of sterilized river sand and grown under daylength-neutral conditions (12 h of light, 100 ± 10 μmol m^−2^ s^−1^, 27 °C; 12 h of darkness, 24 °C) in a growth chamber. Plants were watered with 30 ml of dH_2_O every two days and 20 ml of modified 1/2 Hoagland solution twice a week. Modified 1/2 Hoagland solution was used as described previously with 25 μM phosphate (P) and 1 mM nitrogen (N) [33].

### Experimental design

For Exp. 1, to test the interactions among *V. radiata*, *Ri* (AMF) and *Bj* (rhizobia), experiments were performed with four microbial treatments: control (AMF-, rhizobia-), AMF only (AMF + , rhizobia-), rhizobia only (AMF-, rhizobia +) and both microbes (AMF + , rhizobia +). The experiment followed a randomized complete block design (RCBD) with five batches, and each batch followed a fully factorial design with all four treatments. Each batch contained 160 plants (4 individuals × 10 accessions × 4 treatments). The positions of the pots within the growth chamber were changed every two days to minimize microenvironmental effects. For Exp. 2, we used 20 accessions to evaluate the inoculation efficacy of two rhizobia (*Bj* and *Sinorhizobium fredii* (*Sf*)). Four biological replicates were performed with RCBD. A total of 160 plants (4 biological replicates × 20 accessions × 2 treatments) were used for analysis.

### Plant inoculation with AMF

For Exp. 1, garlic chives (*Allium tuberosum*) was used as the host to maintain the *R. irregularis* spores (IRBV’95 Mycorise ASP; Premier Tech Biotechnologies, Quebec, Canada) in the assays. Briefly, river sand was first autoclaved. Seven-day-old leek seedlings were then inoculated with 0.5 ml of 2 × 10^3^ spores/ml in 1 kg of sterilized river sand. The plants were regularly watered with sterilized water for one week and then with 80 ml of modified 1/2 Hoagland solution (25 μM phosphorus) twice a week. After growing under a 12 h day/night cycle at 22–26 °C for 10 weeks, the leek roots were removed from the river sand, and the sand was allowed to dry for 5 weeks to obtain AMF spore-containing sand. Before the experiment, 5 g of AMF spore-containing sand was used to inoculate 7-day-old leek seedlings in 50 g of sterilized river sand pod without spores for a viability test of the inoculum. After 10 weeks of growing with proper irrigation described above, the roots of leek were stained and observed under a microscope, and the inoculum with more than 90% leek roots infected was used. The same inoculum was used in all the experiments. In the formal experiment, each pot of the AMF treatment group was first filled with 50 g of sterilized river sand without spores. After that, the pots were sprinkled with 5 g of AMF-spore-containing sand as an inoculation layer, and then the rest of the pots were covered with 200 g of river sand without spores.

### Plant inoculation with rhizobia

*B. japonicum* (USDA505) and *S. fredii* (also known as *Ensifer fredii*, USDA205) were obtained from the Bioresource Collection and Research Center of Taiwan (BCRC). Both rhizobia were grown on mannitol-yeast extract media (YEM, 0.01% mannitol, 0.001% yeast extract, 1.87 mM K_2_HPO_4_, 0.81 mM MgSO_4_‧7H_2_O, 1.71 mM NaCl, 1.5% agar) plates at 28 ± 2 °C for 5‒7 days. Cells from single clones were suspended in liquid YEM at 28 ± 2 °C for 5–7 days and centrifuged at 3000 × *g* to obtain cell pellets. The pellets were washed with sterilized water three times and resuspended in water as described in previous studies [34, 35]. The final concentration was adjusted to approximately > 5 × 10^8^ cells/ml. One milliliter of rhizobial solution was then applied to the 7-day-old seedlings. For Exp. 1, only *B. japonicum* was used. For Exp. 2, both rhizobia were used.

### Measurements of plant traits

For both Exp. 1 and Exp. 2, the relative chlorophyll content (RCC) was measured by a chlorophyll meter (SPAD-502, Konica Minolta) at 4, 5, and 6 weeks post-inoculation (wpi). Other plant traits were measured at 6 wpi. Plant height was measured from the soil to the plant apical meristem, and stem width was measured at the widest part. After the sand was removed from the plant roots, the whole plant, shoot, and root fresh weight (FW) were measured. Nodule traits (number and weight) were also separately measured for red and white nodules because mature nodules present a red color and have the ability to fix N, owing to the red color of the active hemoglobin [36]. The rhizobia trait also includes the nodule average FW (total nodule FW/nodule number) and nodule FW ratio (total nodule FW/total plant FW). Leaflets of the first true leaf of all plants were weighed and scanned, and the surface area was measured using ImageJ software. The specific leaf area was calculated by dividing the leaf area by the leaf dry weight (DW). After the samples were dried at 65 °C for 3 days, the plant shoot DW was measured. The water content was calculated as the difference between shoot FW and shoot DW divided by the shoot FW. For Exp. 1, the shoot N or P content was determined by multiplying the shoot DW by the nitrogen or phosphorus content per unit DW. All traits measured in this study are summarized in Table S2.

### Measurement of nitrogen and phosphorus concentrations

For Exp. 1, to measure the nitrogen and phosphorus concentrations, four individuals of the same accession in the same batch were pooled together, and the total number of samples was 200 (10 accessions × 4 treatments × 5 batches), with some missing data. Shoot samples were dried at 60 °C, and a mortar and pestle were used to grind the shoot samples to powder with liquid nitrogen. Each sample (120 mg), together with 90 mg of salicylic acid (C_7_H_6_O_3_, Sigma‒Aldrich), was mixed with 3 ml of sulfuric acid (H_2_SO_4_, 18 M, Echo Chemical). The sample tubes were covered with aluminum foil and placed at room temperature overnight. Afterward, 180 mg of sodium thiosulfate (Na_2_S_2_O_3_, Showa) was added, and the tubes were immersed in a boiling water bath for 3 h and 20 min. For the decolorization reaction, 1.2 ml of 35% hydrogen peroxide (H_2_O_2_, Echo Chemical) was added, and the tubes were placed in a boiling water bath for an hour. Once the tubes had cooled to room temperature, the sample solution was diluted to 30 ml with pure water and filtered through Whatman® qualitative filter paper, Grade 1.

Also, for Exp. 1, to measure the N content, the salicylate method described by Huang et al*.* was modified and used [37]. Briefly, sodium salicylate and sodium hypochlorite buffers were firstly prepared. The sodium salicylate buffer contained 340 mg of sodium salicylate (NaC_6_H_4_(OH)_2_, Merck), 250 mg of sodium citrate (Na_3_C_6_H_5_O_7_, Merck), 250 mg of sodium tartrate (KNaC_4_H_4_O_6_‧4H_2_O, Hayashi Pure Chemical), and 1 mg of sodium nitroprusside (C_5_FeN_6_Na_2_O, Merck) per 5 ml. The sodium hypochlorite buffer included 10 ml of 6% sodium hypochlorite (NaClO) and 0.6 g of sodium hydroxide (NaOH, Showa). The sample buffer was diluted to 1/100 × with pure water, and 20 μl of the sample buffer, together with 90 μl of sodium salicylate and 90 μl of sodium hypochlorite, was transferred to a 96-well plate to react for 30 min. The absorbance was measured at 650 nm. The standard value was obtained by serial dilution of an N standard solution that included 100 mg of ammonium sulfate per liter ((NH_4_)_2_SO_4_, Merck). The P content was detected by inductively coupled plasma optical emission spectroscopy (ICP‒OES), which is a service provided by the NTUAMS ^14^C Dating Lab at National Taiwan University.

### AMF staining and quantification

For Exp. 1, trypan blue was used to stain roots and visually assess AMF colonization. The roots of each plant were cut into fragments of 1.5 to 2 cm and incubated in 10% KOH for 30 min at 96°C. After being washed three times with distilled water, the root samples were placed in 0.3 M HCl for 30 min at room temperature and stained with a 0.1% w/v trypan blue staining solution in a 2:1:1 mixture of lactic acid, glycerol, and distilled water for eight minutes at 96°C. The root samples were de-stained using a solution of 1:1 glycerol and 0.3 M HCl [38]. For each plant, 30 randomly selected root sections on slides were observed using a microscope (Olympus BX51). AMF colonization levels were quantified with a modified gridline intersect procedure as previously described [39].

### Statistical analysis

All data were analyzed with mixed-model analysis of variance (ANOVA) in JMP (Version 16, SAS Institute Inc., Cary, NC, USA). For Exp. 1, in all following analyses regarding the tripartite interaction experiments, “batch” was used as a random effect to control for variation among the experimental batches. “Accession” was used as a random effect nested within the fixed effect “subspecies”, and fixed effects also included “AMF” and “rhizobia”, as well as the subspecies-AMF-rhizobia interactions (model 1, Table S3). To investigate the change in leaf RCC over time, we used “subspecies”,”rhizobia”,”AMF,” and “week” and their interactions as fixed effects and “accession” nested within subspecies as a random effect (Table S4). To investigate rhizobia fitness, we used mungbean plants inoculated with rhizobia (Table S5). The model was almost the same as Model 1, except that nodule traits (nodule number, nodule FW, nodule average FW, and nodule FW ratio) were used as response variables, and the rhizobia effect was removed (Table S5). To investigate AMF fitness, we used mungbean plants inoculated with AMF (Table S6). The model was almost the same as Model 1, except that the AMF colonization traits (vesicle, arbuscular, and AMF colonization) were used as response variables, and the AMF effect was removed (Table S6). The significance of pairwise contrasts was tested with Tukey’s honestly significant difference (Tukey-HSD) test with an α of 0.05. To determine the correlation between total nodule FW and plant traits, we followed Model 1 but used “total nodule FW” instead of the “rhizobia” treatment effect, and only data from mungbean plants inoculated with rhizobia were used (Table S7). Heritability was estimated separately for each treatment (control, AMF, rhizobia, and AMF + rhizobia). Here, “accession” was used as a random effect nested within the random effect “subspecies”, and “batch” was also used as a random effect (Table S8). The heritability was calculated by the following equation:

$$\left({\text{Var}}_{\text{Subsp}}+{\text{Var}}_{\text{Acc}}\right)/{\text{Var}}_{\text{All}},$$

where Var_Subsp_ is the variance component of subspecies, Var_Acc_ is for accession, and Var_All_ is the sum of all variances. The magnitude of trait divergence *Q*_*ST*_ was calculated as follows:

$${\text{Var}}_{\text{Subsp}}/\left({\text{Var}}_{\text{Subsp}}+ {\text{Var}}_{\text{Acc}}\right)$$

In Exp. 2, to test the affinity of two rhizobia species for 20 mungbean accessions, we used the “treatment” effect, which represented inoculation with *B. japonicum* or *S. fredii*; the “group”, which represented a fixed effect for the 4 genetic groups; and the “accession” nested within group, which represented a random effect (Table S9).

## Results

### Effects of interactions on plants

To evaluate plant fitness under rhizobia-AMF interactions, we selected five wild (*V. radiata* subsp. *sublobata*) and five cultivated (*V. radiata* spp. *radiata*) accessions, including at least one accession in each cultivar population [13] and wild accessions from South Asia to Australia, representing worldwide diversity (Fig. S1A). All traits differed significantly among genotypes (with significant accession random effects according to mixed-model ANOVA). The heritability was greater for traits related to plant weight (Table S8). The cultivars were generally sturdier and heavier than the wild mungbean plants (Fig. S2A and S2B), which is supported by the high *Q*_*ST*_ across treatments (Table S8).

**Fig. 1:**
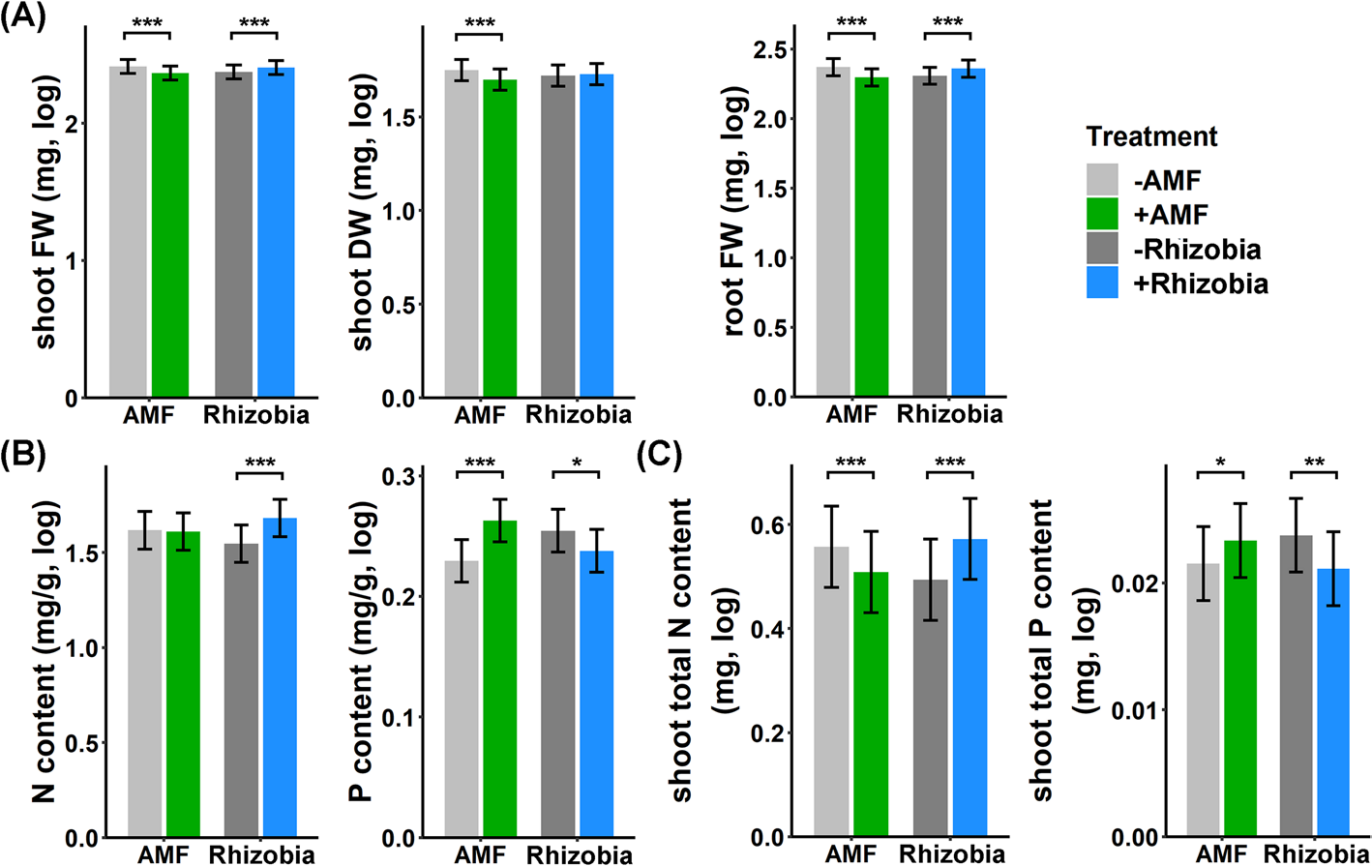
Main effects of *Ri* and *Bj* on plant traits. **A** Shoot fresh weight (FW, mg), shoot dry weight (DW, mg) and root FW (mg) after log10 transformation. **B** Unit element content in mungbean (mg/g) after log10 transformation. **C** Total element content in shoots (mg) after log10 transformation. Color represents inoculation with or without *Ri* and *Bj*, light grey: without *Ri*; green: with *Ri*; grey: without *Bj*; blue: with *Bj*. Color bars represent the least squares mean, and the error bars represent standard error (± SE). Asterisks denote statistical significance of the main effect with or without microorganisms. * represents *P* < 0.05. ** represents *P* < 0.01. *** represents *P* < 0.001

On average, the presence of *Bj* increased plant weight, while *Ri* had the opposite effect (Table S3 and Fig. 1A). The effect of *Bj* on plants was still significant after excluding nodule weight (pure root FW, Table S3). Interestingly, the heritability of plant weight-related traits was greater under *Bj* than under *Ri*, suggesting that the beneficial effect of *Bj* might be specific to different plant accessions, while *Ri* had a more general detrimental effect (Table S8).

Regarding the element content per unit plant tissue (Table S3, Fig. 1B), *Bj* significantly increased the N content, and *Ri* had no effect. Interestingly, while *Ri* increased the P content, as expected, *Bj* appeared to decrease P in the plant. By multiplying the unit element content by plant weight, we observed the same trend except that *Ri* significantly decreased the total plant N content (Fig. 1C), likely due to its effect on decreasing plant weight (Fig. 1A).

**Fig. 2:**
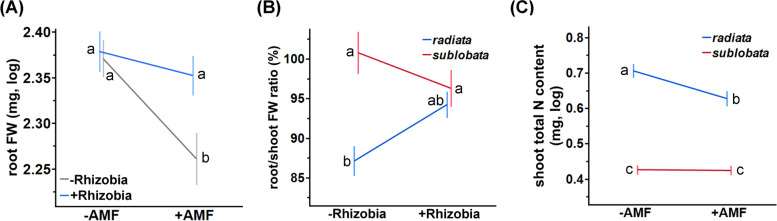
The interaction effects among mungbean subspecies, *Ri*, and *Bj* on plant traits. **A** Significant *Ri* by *Bj* interaction effects on root FW. **B** Significant *Bj* by mungbean subspecies interaction effects on plant root/shoot FW ratio. **C** Significant *Ri* by mungbean subspecies interaction effects on shoot total N content. Y-axis values represent the least squares mean, and the error bars represent ± SE. Different letters on the bars indicate significant differences (*P* < 0.05) under Tukey’s HSD test

Considering the synergistic effects among *Bj*, *Ri*, and mungbean subspecies, significant interactions between *Bj* and *Ri* were mainly observed for root FW-related traits (Table S3 and Fig. 2A). While *Ri* significantly decreased root FW, the presence of *Bj* eliminated such negative effects of *Ri*. A similar trend was also observed for heritability, where the presence of *Bj* restored the decreased heritability under *Ri* treatment (Table S8). This pattern was not caused by extra weight from nodules, as this interaction effect was also observed in the root FW excluding nodules (Table S3). Interestingly, the root/shoot FW ratio was also affected by the interaction between *Bj* and the mungbean subspecies. The cultivars had much lower underground resource allocation than the wild progenitors, but the presence of *Bj* restored the root/shoot FW ratio in the cultivars (Table S3, Fig. 2B). A similar pattern was also observed for the pure root/shoot FW ratio without nodules, which indicated that this ratio was not affected by nodule weight. In other words, underground resource allocation in cultivars appeared to be more responsive to symbionts. Regarding the subspecies*Ri interaction, we found that the presence of *Ri* significantly reduced the shoot N content in the cultivars but not in the wild subspecies (Fig. 2C), indicating a greater sensitivity of the negative effect in some cultivars.

**Fig. 3:**
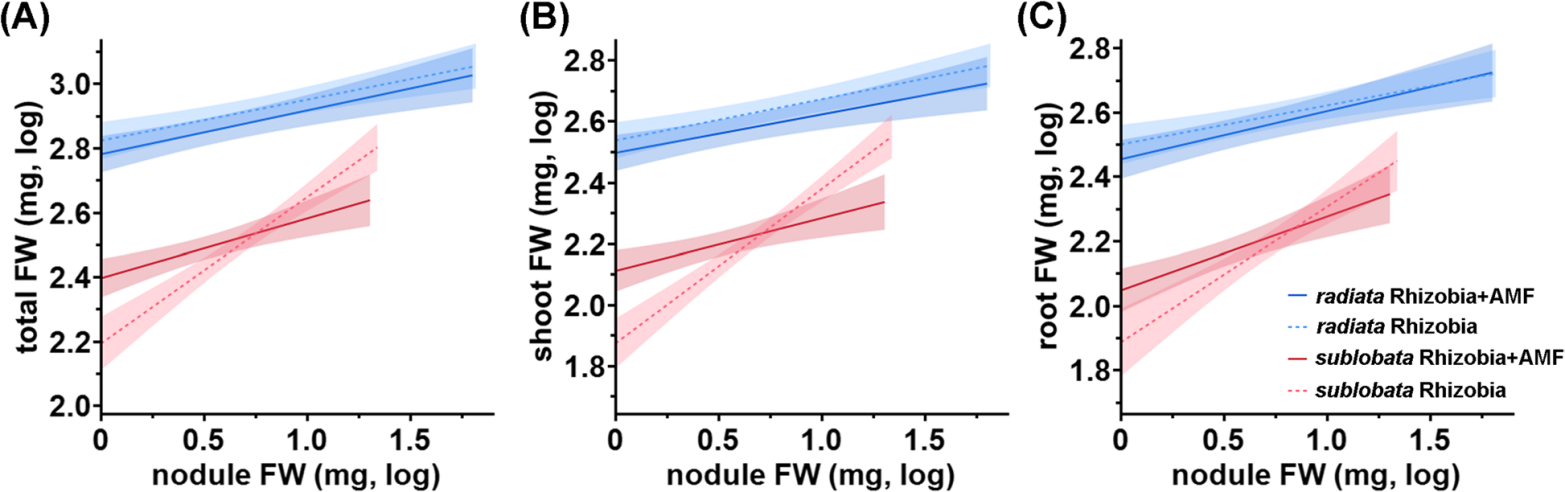
The distinct effects of mungbean subspecies and *Ri* on the relationship between nodule weight and plant traits. **A** Nodule FW effects on total FW. A significant difference of the nodule FW effect with/without AMF (AMF*nodule FW) on total FW was observed in *sublobata*, but such effect was insignificant in *radiata*. **B** Nodule FW effects on shoot FW. The patterns of statistical significance were similar to (**A**). **C** Nodule FW effects on root FW. No significant AMF*nodule effect exists in either subspecies. Light red dash line: *sublobata* under *Bj*-only treatment; red line: *sublobata* under *Bj* + *Ri* treatment; light blue dash line: *radiata* under *Bj*-only treatment; blue line: *radiata* under *Bj* + *Ri* treatment. The lighter color range according to every line represents 95% CI of the line values. The full results of the analysis of variance are in Supplementary Table S3

The above analyses investigated the effects of the *Bj* and *Ri* treatments, and we noted that the mungbean accessions exhibited strong differences in nodulation efficiency (high heritability of nodule traits, Table S8) but not in terms of AMF colonization. To control for such an effect, we performed similar analyses only with plants inoculated with *Bj*, using nodule FW as a linear fixed effect (Table S7). Nodule FW had strong positive correlations with most traits, confirming that *Bj* positively affected plant performance (Fig. 3, Table S7 and Fig. S3). The interaction between *Ri* and nodule FW had significant effects on shoot total N content, total FW, and shoot FW but not root FW, whereas the presence of *Ri* significantly attenuated the positive effects of nodule FW on these traits (Fig. 3 and Table S7A). The results indicate that the interaction affects only the shoot system of the plants. Although the three-way interactions among subspecies, AMF, and nodule FW were not statistically significant, probably due to sample size, wild mungbean was more responsive to *Ri*-nodule FW interaction effects when we performed the analyses separately for each subspecies (Fig. S3 and Table S7B, C). This is reflected in Fig. 3, where the plant-nodule relationships in *sublobata* had different slopes with or without *Ri*. Therefore, total nodule FW had a positive effect on plant growth (reflecting the “fitness alignment” in Afkhami et al. 2021), and *Ri* attenuated such positive effects on more traits in the wild mungbean than in the cultivars.

**Fig. 4:**
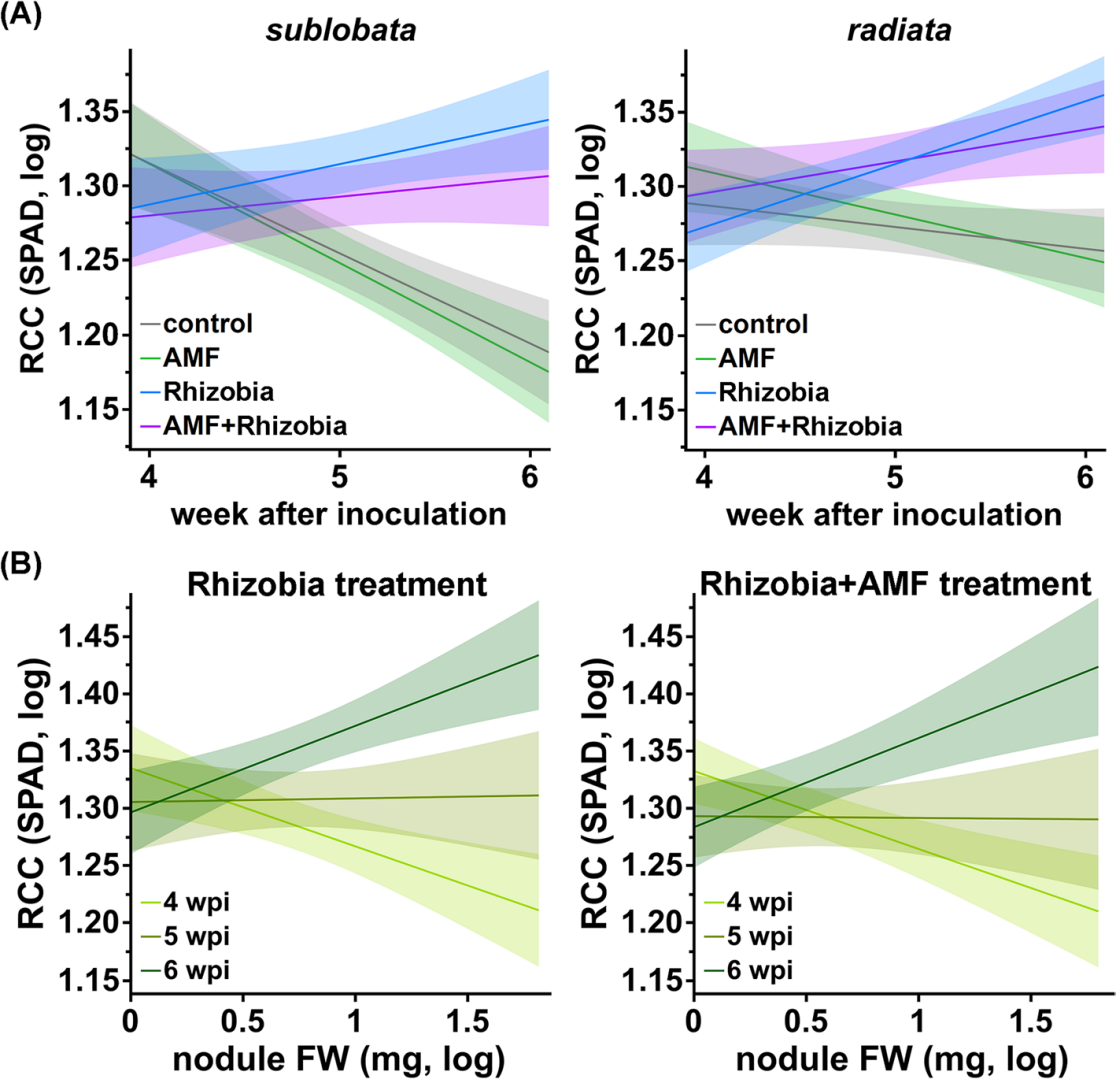
Effect of *Ri* and *Bj* on the relative chlorophyll content (RCC) of plant leaves. **A** Comparison of relative chlorophyll content (RCC) through time in mungbean leaves under four treatments. Color of four treatments, grey: control; green: *Ri* treatment; blue: *Bj* treatment; purple: *Ri* + *Bj* treatment. **B** Relationship between RCC and total nodule weight. SPAD: values measured by the Soil Plant Analysis Development (SPAD) chlorophyll meter. Light green: 4 weeks post-inoculation (wpi); green: 5 wpi; dark green: 6 wpi. Lines are the linear regression lines. The shades represent 95% confidence interval (CI) of the line values

N is one of the most important nutrients for photosynthesis, and there is a strong positive correlation between N supply and chlorophyll content [40]. Consistently, there was an opposite trend of relative chlorophyll content (RCC) change across time with or without *Bj* (significant rhizobia*week interaction, *P* < 0.0001; Fig. 4A and Table S4). The effects of *Bj* treatment on RCC increased from 4 to 6 wpi (*P* values were 0.0241, 0.0003 and 0.0001, respectively; Table S3). This time-dependent effect was especially strong in the wild accessions (Fig. 4A and Table S4), given that the *Bj* interaction had a significant effect on the subspecies at 6 wpi but not at other time points (Table S3); therefore, the Subspecies*rhizobia*week interaction effect was significant (*P* = 0.0397). Interestingly, we noticed that the relationship between nodule weight and RCC reversal depended on the timepoint (Fig. 4B and Table S7). This phenomenon is probably due to nodules acting as resource sinks in the early stages of symbiosis, whereas their N-fixation effects become more pronounced later. This was consistent with the results showing that at 4 wpi, plants without *Bj* treatment generally had greater RCC than those inoculated with *Bj*, and the trend was reversed later (Fig. 4A and Table S3). Therefore, *Bj* might benefit plants by increasing RCC, possibly affecting photosynthesis efficiency, and wild mungbean might be more dependent on this effect than cultivars.

### Effects of interactions on symbiotic microbes

**Fig. 5:**
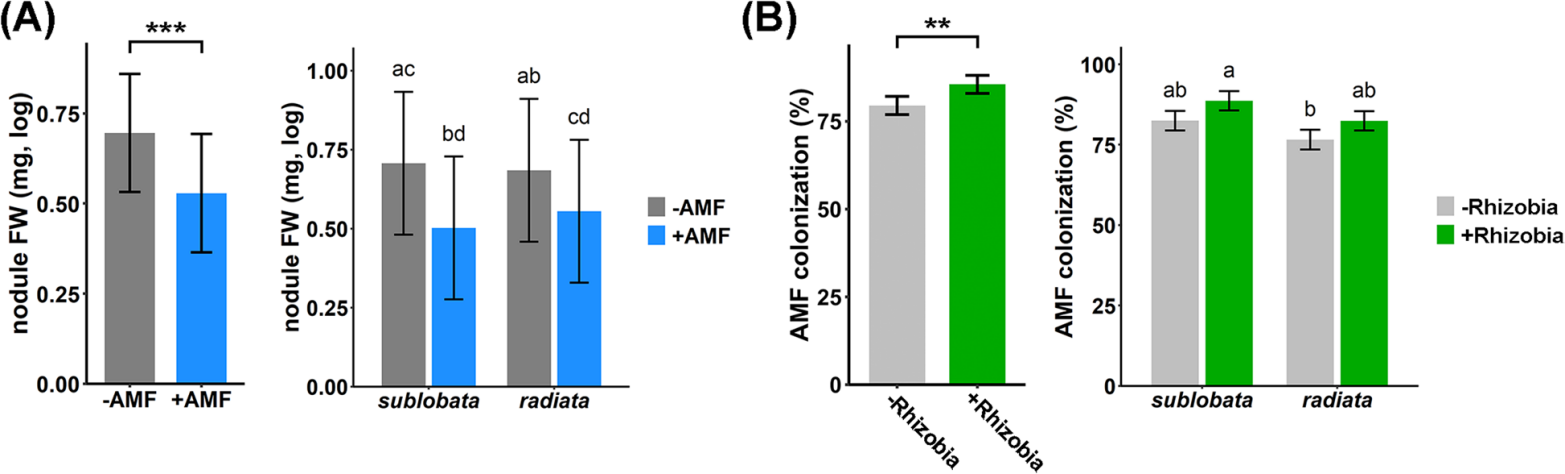
Effects of the presence of *Ri* and *Bj* on each other. **A** Total log10 nodule FW in plants treated with *Bj*, plotted combined or separately for two mungbean subspecies. **B** *Ri* colonization in plants treated with *Ri*, plotted combined or separately for two mungbean subspecies. Color bars represent the least squares mean, and the error bars represent ± SE. Different letters on the bars indicate significant differences (*P* < 0.05) under Tukey’s HSD test

The tripartite interactions among plants, rhizobia, and AMF may affect not only plant phenotypes but also the fitness of symbiotic microbes. In general, AMF decreased the fitness of rhizobia: total nodule FW and nodule number were significantly reduced by the presence of *Ri* (Fig. 5A, Table S5, Fig. S4E and Fig. S5A). The negative effect of *Ri* appeared to act on nodule number but not the average FW of each nodule (Table S5 and Fig. S4F).

Plant subspecies have little effect on nodule traits (Table S5 and Fig. 5A), but large variation exists among plant accessions, reflected by the high heritability but low *Q*_*ST*_ in nodule traits (Table S8). While most wild accessions could form certain numbers of nodules with *Bj*, high variation in nodule affinity was observed among the cultivars (Fig. S5). Since the color of the nodule might reflect its functionality and the red nodules might have a greater ability to fix N [41], we separated the nodules into red- and white-colored groups. The results showed that the red nodule-related traits had patterns similar to those of the total nodule (Fig. S4A-D and Fig. S6).

AMF fitness could be approximated by the proportions of AMF colonization, arbuscules, and vesicles within plant roots. The presence of *Bj* significantly enhanced *Ri* colonization but not the proportions of arbuscules or vesicles (Fig. 5B, Table S6 and Fig. S7). Mungbean accessions showed similar proportions of *Ri* colonization, arbuscules and vesicles, indicating that *Ri* had no obvious host specificity (Fig. 5B and Fig. S7). These results were also reflected by the low heritability (Table S8).

### Rhizobia exhibited distinct host specificity to mungbean accessions

Given that *Bj* formed drastically different numbers of nodules among accessions (Fig. S5 and S6), we further investigated the affinity between two rhizobia species (*B. japonicum Bj* and *S. fredii Sf*) and a broader range of mungbean accessions. A total of 20 accessions were used, with five from each of the four genetic groups [13]: *sublobata*, South Asian *radiata*, Southeast Asian *radiata*, and North Asian (East Asian + Central Asian) *radiata* (Table S1).

**Fig. 6:**
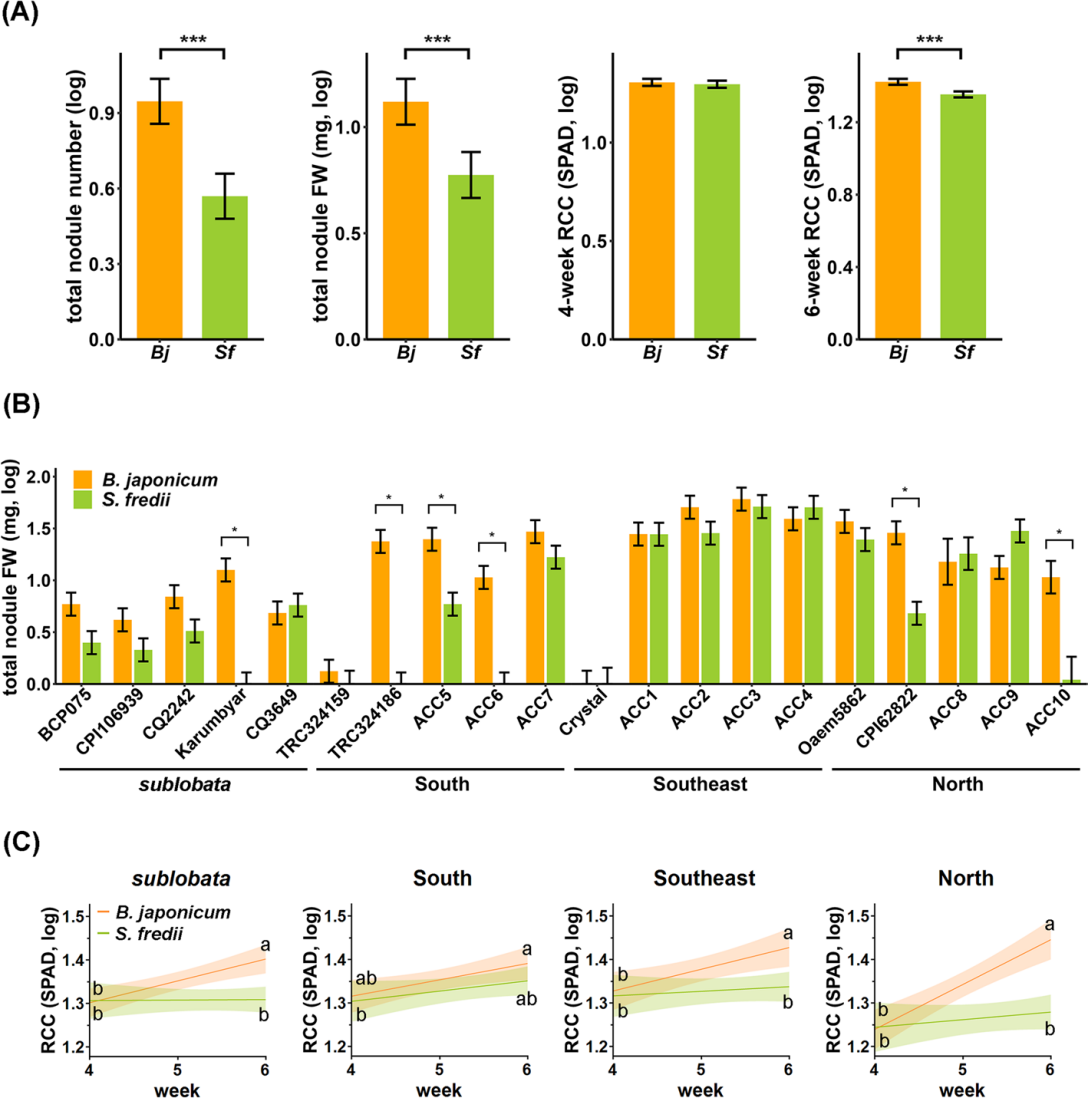
Effects of plant accessions on the nodulation efficacy of *B. japonicum* (*Bj*) and *S. fredii* (*Sf*) and their respective effects on plants. **A** Comparisons of traits under two rhizobia treatments. *Bj: B. japonicum; Ef: S. fredii*; *** represents *P* < 0.001 in Student’s t-test. **B** Total nodule FW of the 20 mungbean accessions, showing differences among plant accessions and rhizobia species. Asterisks indicate significant differences (*P* < 0.05) under Tukey’s HSD test. **C** Comparison of RCC in mungbean inoculated with two different rhizobia species. Each panel represents RCC changes through time in one mungbean population. The North population had the strongest positive relationship between week and RCC with the presence of *Bj*, while the South population showed the weakest relationship with *Bj*. The lighter color range around every line represents 95% CI. Lines represent the analyses using all three time points (4, 5, and 6 weeks) and time as a continuous variable. Letters indicate significant differences (*P* < 0.05) between 4 and 6 weeks under Tukey’s HSD test in a statistical model, using only these two time points and time as a binary categorical variable. Color represents two treatments, orange: inoculation with *Bj*; green: inoculation with *Sf*

In general, the two rhizobia species had different affinities, with *Bj* resulting in a greater nodule number and weight than *Sf* (Fig. 6A and Table S9). Although mungbean genetic groups have highly different weight-related traits according to previous observations [13], they do not differ significantly in terms of nodule traits. Instead, the distinct rhizobia affinity is accession-specific (Fig. 6B).

For plant health, in terms of relative chlorophyll content (RCC), while the two rhizobia species generated low and similar RCCs at 4 wpi, *Bj* resulted in a greater 6-wpi RCC than *Sf* (Fig. 6C and Table S9). Consistent with our previous results showing that the beneficial effects of rhizobia became more pronounced in later developmental stages, *Bj* appears to have a greater contribution to plant health. Mungbean was initially domesticated in South Asia and first spread to Southeast Asia, where it finally reached the northern parts of Asia [13]. Here, we found that the ability of *Bj* to promote plant health increased during the course of domestication (Fig. 6C). On the other hand, *Sf* resulted in a similar RCC regardless of wpi, and the pattern remained similar across mungbean genetic groups, in stark contrast to *Bj*. In summary, *Bj* has a greater affinity for mungbean and promotes chlorophyll content more efficiently, and this beneficial effect appeared to increase during the course of mungbean domestication, which is consistent with the ancient Chinese agricultural literature Qimin Yaoshu (齊民要術, 544 AD) emphasizing mungbean’s value as green manure in northern China.

## Discussion

### Tripartite symbiosis among rhizobia, AMF, and mungbean

**Fig. 7:**
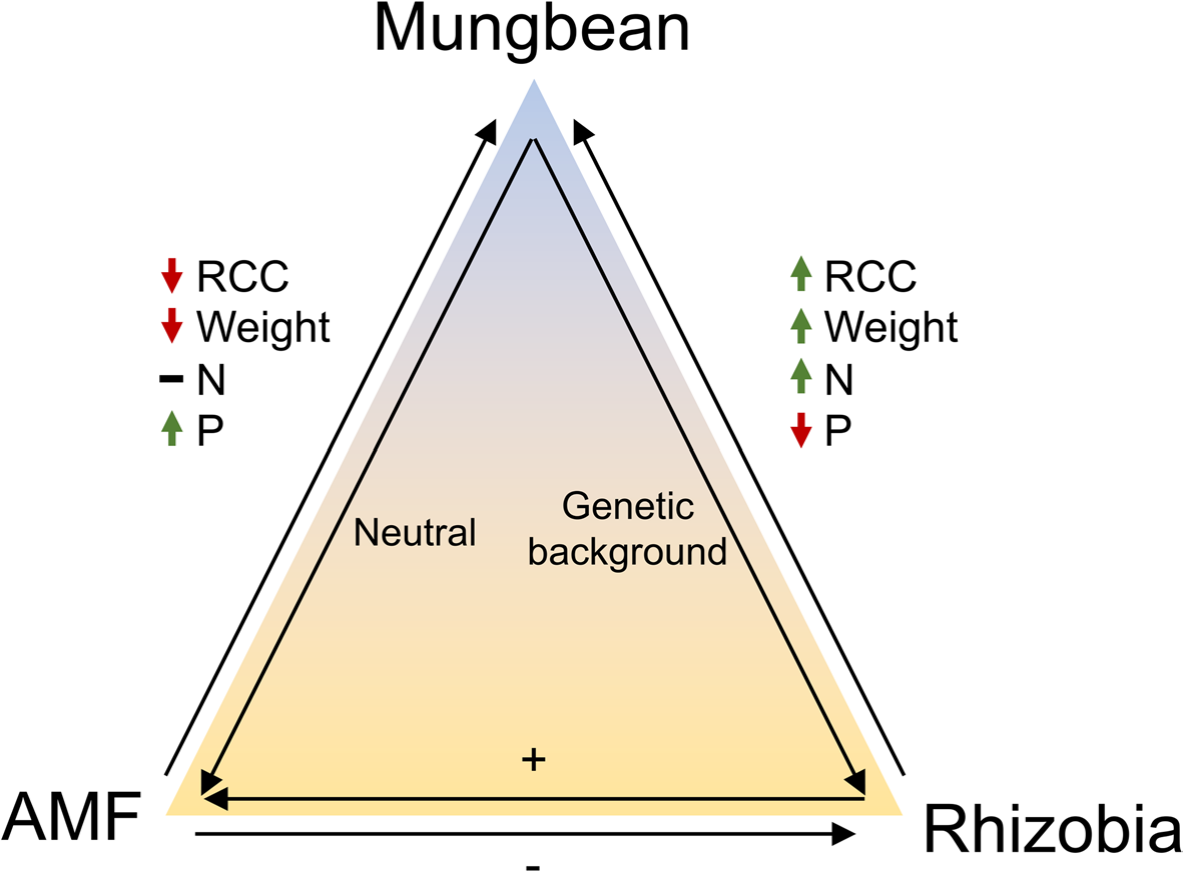
Summary of the tripartite symbiosis among mungbean, rhizobia and AMF. Mungbean genetic background affected rhizobia but not AMF colonization. Rhizobia generally had positive effects on the two other parties except for decreasing phosphorus content in plants. AMF generally had negative effects on the two other parties except for increasing phosphorus content in plants

The benefit of symbionts can vary depending on the environment and timing. Therefore, to reduce the use of chemical fertilizers, it is essential to understand the interactions within the tripartite symbiosis involving AMF, rhizobia, and the host. Additionally, different cultivars may exhibit diverse responses influenced by their genetic factors to symbiotic microbes, leading to distinct preferences for symbiosis. Under adverse conditions, how plants interact with these symbiotic microbes could be one of the most important issues for legume agriculture. To date, most recent studies have focused on the relationships of a single plant accession with few rhizobia/AMF species, and only a few large-scale studies have investigated multiple within-species accessions and multiple symbiotic microbes. Therefore, a study integrating multiple accessions and microbes is needed because the host’s genetic background could be diverse and affect symbiotic outcomes. In this study, we selected five accessions from each of the wild (*V. radiata* ssp. *radiata*) and cultivated (*V. radiata* ssp. *sublobata*) mungbean subspecies, representing worldwide genetic variation in this species (Table S1). We aimed to investigate the outcomes of bilateral/tripartite interactions involving mungbean subspecies, AMF, and rhizobia. To minimize the potential influence of various P and N supplies, we performed the entire experiment under the same environment with fixed low P (25 μM) and low N (1 mM) supplies. Low P and low N were used because previous studies showed that sufficient P or N supplies might decrease the symbiotic efficiency between the microbe and the host [32, 42], which could introduce uncertainty in the evaluation of the nutrient uptake ability of the microbes. The results indicate that under the experimental conditions, *Bj* was a more beneficial partner of mungbean than *Ri*, although the benefit of *Bj* depends on the specific mungbean accession. Under the tripartite interaction, the nodule number and FW of *Bj* decreased when mungbean was inoculated with *Ri*, whereas the colonization rate of *Ri* increased when mungbean was co-inoculated with *Bj*. Overall, during the timelapse of the experiment, the presence of *Bj* increased the FW, RCC, and N in the host, but decreased P. In contrast, *Ri* negatively affected FW and RCC, but increased P (Fig. 7).

Among the hypotheses on plant–microbe interactions, the latest hypothesis stands that short-term competition in the biosphere tends to evolve into a long-term cooperative system, increasing biodiversity and decreasing ecological conflicts, called “cooperative equilibrium [43].” When the environment changes, especially when the N:P:C ratio is altered, mutualism might become parasitism in plant–microbe interactions [3, 11, 44] until a new equilibrium is formed. The previous studies showed that under certain degrees of available N and P or under natural field conditions, AMF is a beneficial partner to mungbean in tripartite symbiosis [9, 27–30]. However, under N- and P-limited conditions, our mungbean-rhizobia-AMF system did not conform to the previous results. The cooperative equilibrium was broken under this condition. Although the existence of *Bj* benefited the mungbean, *Ri* reduced the biomass of the host while increasing the host’s P content. The symbiosis between *Ri* and mungbean shifted from mutualism to parasitism, showing the nutrient tradeoff symptoms between these two organisms. Therefore, under N- and P-limited conditions, *Bj* could be a stable partner of mungbean with fewer ecological conflicts, whereas the mutualism between *Ri* and mungbean largely depended on the existing nutrients in the environment. Further study is necessary to address the complexity of outcomes in this tripartite system.

Wild and cultivated mungbean subspecies exhibit notable distinctions in their phenotypic and genetic characteristics, which may lead to varying responses to symbiotic interactions. Wild mungbean generally appears to be more plastic, exhibiting stronger negative and positive impacts from AMF and rhizobia, respectively. For example, AMF treatment reduces the positive effects between plants and the rhizobia fresh weight, and the effect appears to be much stronger in the wild than in cultivated mungbean (Fig. 3). Although the lack of rhizobia decreased the leaf chlorophyll content over time, the effect also appeared to be stronger in the wild group than in the cultivar group (Fig. 4A). In summary, the findings suggest that wild mungbean varieties may exhibit greater sensitivity to microbes, while cultivated mungbean subspecies could display reduced responsiveness to these microorganisms. Moreover, cultivated mungbean demonstrated enhanced nutrient uptake efficiency, as evidenced by their significantly greater biomass and higher total N and P contents (Fig. 3 & Fig. S3).

Previous studies have shown that dual inoculation of AMF and rhizobia could promote legume growth in *Medicago truncatula* [8], *V. radiata* [9], *Glycine max*, *Phaseolus mungo*, *Cicer arietinum*, *Lens culinaris* and *Phaseolu mungo* [12]. Here, we found that the positive *Bj* effect and negative *Ri* effect are not simply additive: strong interaction effects exist for many plant traits. Regardless of whether we modeled the effect of rhizobia in terms of qualitative treatment effect or quantitative nodule weight, we observed similar trends in which *Ri* had detrimental effects on plant weight, but rhizobia ameliorated these negative effects (Fig. 3 & Fig. S3). Afkhami et al*.* [8] modeled the relationship between plant fitness and rhizobia fitness (nodule number), calling this positive relationship the “fitness alignment” between symbionts. The authors showed that AMF could increase the responsiveness of *M. truncatula* to the beneficial effects of rhizobia, while our study showed that *Ri*, when it had an overall negative effect, further decreased the responsiveness of *V. radiata* to the benefits of *Bj*. In addition, while Afkhami et al*.* [8] focused on pod number, we showed that different plant parts have distinct effects. When focusing on those plants inoculated with *Bj* and modeling nodule weight as *Bj* fitness, *Ri* had a negative impact on the relationship between plants and *Bj* only in plant shoots but not roots, and the effect appeared to be specific to the wild subspecies (Fig. 3 & Table S7). In this experiment, *Ri* showed a characteristic position in the mutualist-parasite spectrum, where it increased the P content but reduced the biomass of both the plants and *Bj*. In contrast to previous studies, here, we show that the effect is not universal but could differ among genetic backgrounds and plant parts.

Noticeably, we only used *Ri* as an AMF in this study, and it remains unclear whether the outcome will be similar to that of other AMFs. Although *Ri* has been reported to increase the biomass of mungbean [27], it showed opposite effects in the conditions we used. A previous study also reveals that the mixture of AMF inoculum (a mixture of five species of AMF, including *Ri*) has no significant effects on the mungbean, while the dual inoculation of rhizobia and AMF increases the yield [28]. It is unclear whether the different AMFs have different mutualist-parasite spectrums to the mungbean, or whether the results we observed could apply to the field soil conditions (we used pots with sterile sands in our system). In addition, AMF has been reported to benefit plant hosts with different growth periods [45]. The syngeneic effects of dual inoculation of AMF and rhizobia were observed after 11 wpi in mungbean in the previous works [29, 30]. In our experiments, we only recorded traits as long as 6 wpi, and it is unknown if *Ri*’s effects would become positive at the prolonged time points. Further studies are needed to clarify these issues.

### Fitness of rhizobia species toward various mungbean accessions

In our study, we found that *Bj* had various effects on different mungbean accessions, but how does *Bj* affect other rhizobia species? Among the dominant genera of rhizobia, *Sinorhizobium* and *Bradyrhizobium* are classified into a fast-growing rhizobia and a slow-growing rhizobia, respectively [16]. In previous studies, *Bj* has been shown to be more dominant than *Sf* when two rhizobia species co-inoculate the same host plant [17–21, 35]. On the other hand, compared with *Bj*, *Sf* could form more nodules on soybeans at the same bacterial concentration [35]. Our studies showed that *Bj* formed more nodules than *Sf* (Table S9). Additionally, compared with *Sf*, *Bj* promoted more RCC in mungbean and was less specific to host genotypes, indicating that mungbean might have a more efficient symbiosis with slow-growing *Bj* than with fast-growing *Sf*. Our data showed that many accessions had strong symbiosis with *Bj* but had no or very low affinity for *Sf* (Fig. 6B). Thus, these accessions could be candidates for studying the mechanism of symbiosis between the host and rhizobia.

## Conclusions

Our study revealed that tripartite symbiosis does not unconditionally benefit all parties under all conditions, and the complex relationship does not conform to one universal outcome. Instead, different parts of the tripartite relationship could have distinct results (Fig. 7). This provides an interesting starting point to further investigate their complex interactions and the role of each party in the tripartite symbiosis. In short, our study expands the knowledge of the effects of the tripartite symbiosis of *V. radiata* at the subspecies/accession level, and the investigation of symbiosis between two rhizobia and mungbean accessions provides useful information on the combination with the highest efficiency.

### Supplementary Information

Supplementary Material 1

## Data Availability

Data will be made available on request from the corresponding author.
