# Supplementary material for: The costs and benefits of symbiotic interactions: variable effects of rhizobia and arbuscular mycorrhizae on Vigna radiata accessions
Source: BMC Plant Biol. 2024 Aug 15;24:780. doi: 10.1186/s12870-024-05488-5 (PMC11325573; doi:10.1186/s12870-024-05488-5)
Supplement: Supplementary file 1 — Supplementary Material 1 [file 12870_2024_5488_MOESM1_ESM.pdf]

Supplementary Information

The costs and benefits of symbiotic interactions: variable effects of rhizobia and arbuscular mycorrhiza on *Vigna radiata* accessions

Chih-Cheng Chien[1]*, Shang-Ying Tien[2], Shu-Yi Yang[2], Cheng-Ruei Lee[1,2]*

[1]Institute of Ecology and Evolutionary Biology, National Taiwan University, Taipei, Taiwan
[2]Institute of Plant Biology, National Taiwan University, Taipei, Taiwan

***Correspondence:**
Cheng-Ruei Lee
chengrueilee@ntu.edu.tw
Chih-Cheng Chien
ccchien1983@ntu.edu.tw

This file includes:

Supplementary Fig. S1 to S7.

Supplementary Table S1 to S9.

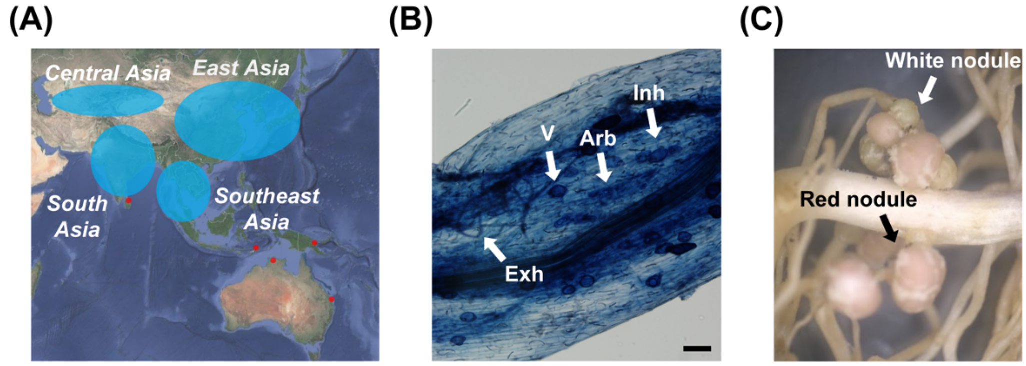

Fig. S1 Plant materials and phenotypes at 6 weeks post-inoculation (wpi). (A) Origins of accessions used in this study. Red dots represent *V. radiata* spp. *sublobata*. Blue circles represent *V. radiata* spp. *radiata* populations. (B) Observation of arbuscular mycorrhizal fungi (AMF) structure (Crystal accession, 200X). Exh: extraradical hyphae; Inh: intraradical hyphae; V: vesicle; Arb: arbuscule. Bar: 100 μm. (C) The morphology of nodules (accession: ACC9).

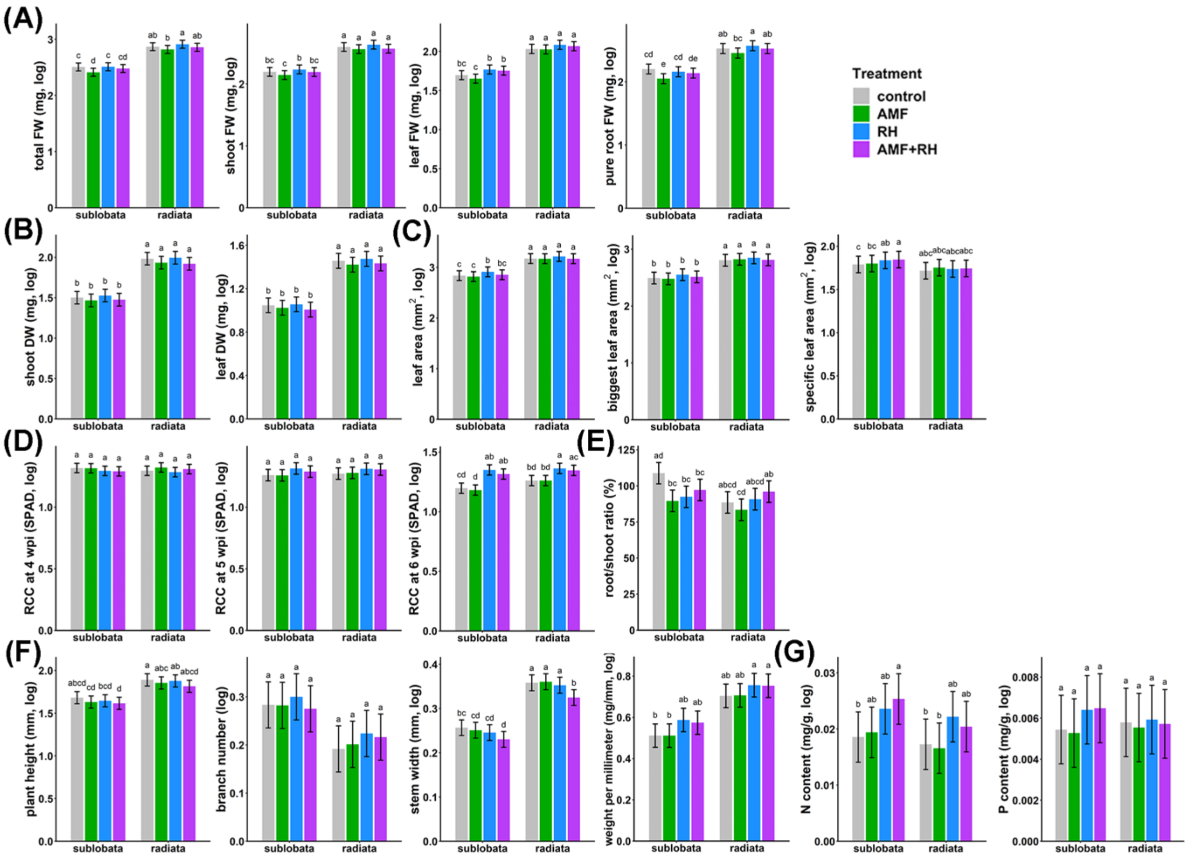

Fig. S2 Comparison of other plant traits under four treatments. (A) Plant fresh weight under four different treatments (control, *Ri*, *Bj*, *Ri* + *Bj*). (B) Plant dry weight, (C) leaf area-related traits, (D) RCC at different week-post-inoculation (wpi) under four treatments, (E) Root/shoot fresh weight (FW) ratio, (F) biomass-related traits, and (G) N and P content (mg/g) under different treatments. Bars represent the least squares mean, and error bars represent standard error (±SE). Different letters on the bars indicate significant differences ($P < 0.05$) under Tukey's HSD test.

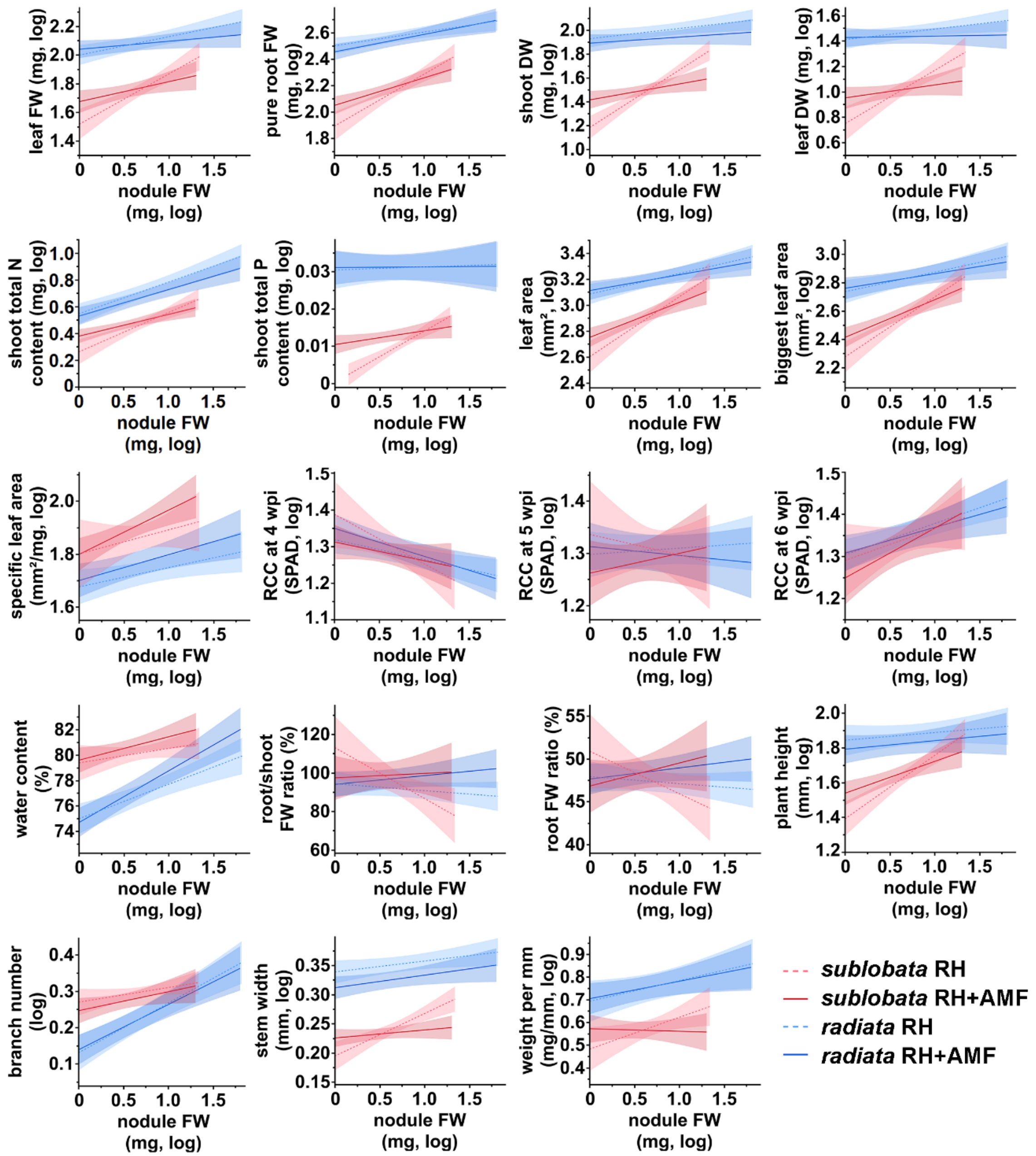

Fig. S3 Effect of mungbean subspecies and AMF treatment on the relationship between nodule weight and plant traits. Light red dash line: *sublobata* under *Bj*-only treatment; red line: *sublobata* under *Bj*+*Ri* treatment; light blue dash line: *radiata* under *Bj*-only treatment; blue line: *radiata* under *Bj*+*Ri* treatment. The lighter color range around every line represents 95% CI.

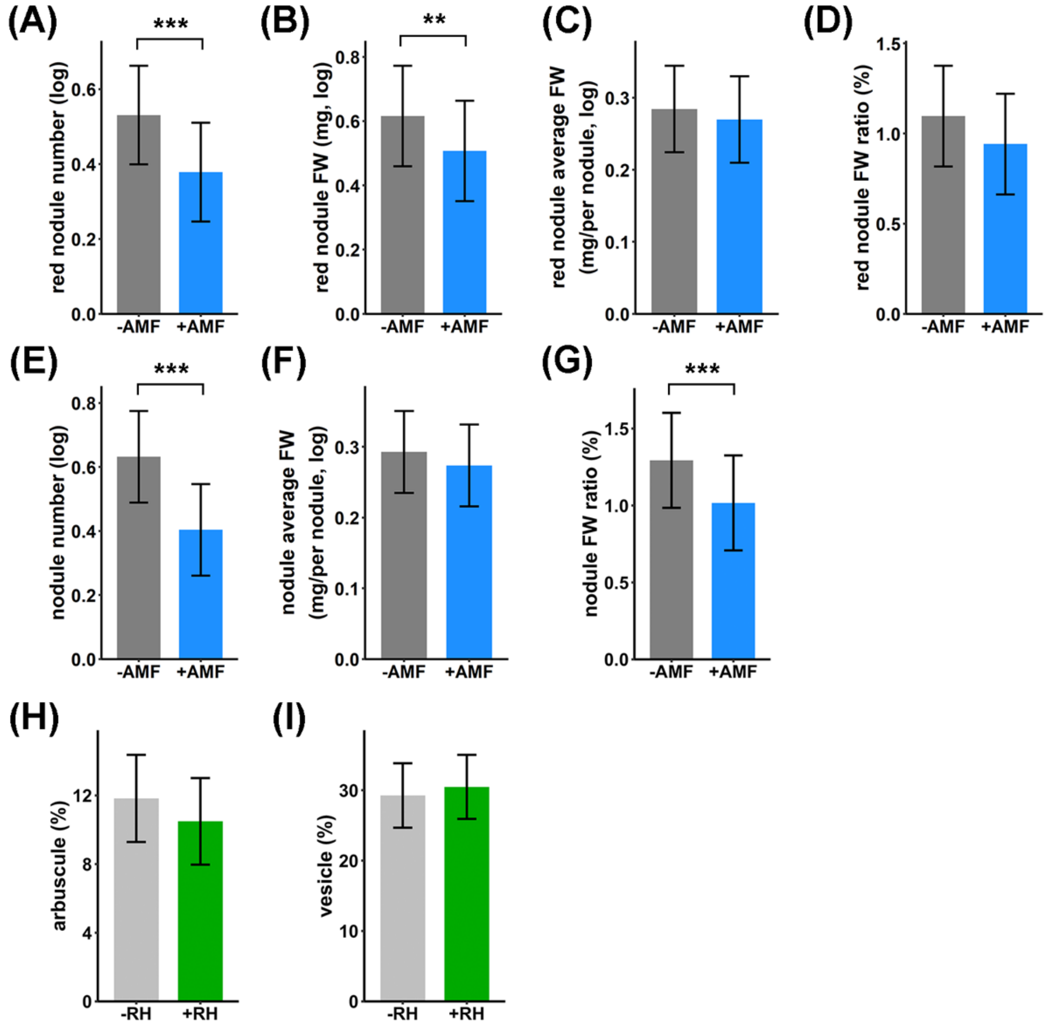

Fig. S4 Comparison of RH-related and AMF-related traits in mungbean with *Bj*, *Ri* and *Bj* + *Ri* treatments. (A-G) Comparison of nodule-related traits with/without *Ri* under the presence of *Bj*. (H, I) Comparison of AMF-related traits with/without RH under the presence of *Ri*. Bars represent the least squares mean of the data after log transformation, and error bars represent ±SE. The asterisks represent statistical significance in analysis of variance (*: $P < 0.05$; **: $P < 0.01$; ***: $P < 0.001$).

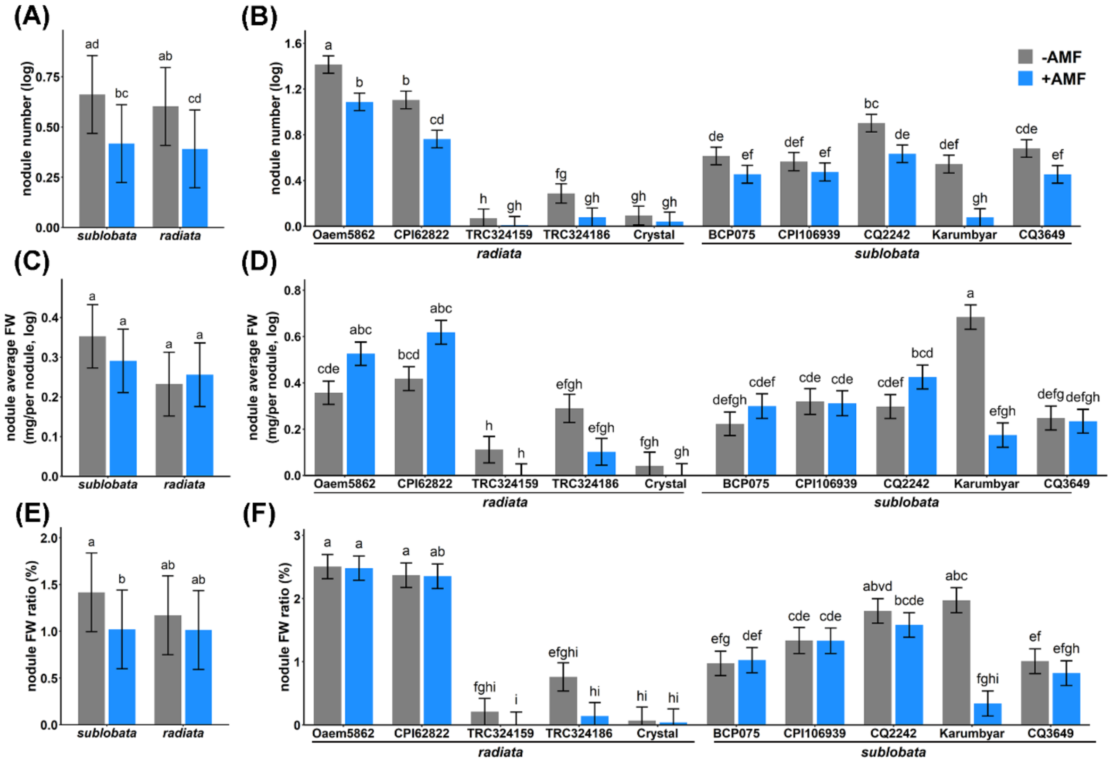

Fig. S5 Comparison of nodule-related traits in mungbean subspecies and accessions with *Bj* and *Bj* + *Ri* treatments. (A, C, E) Comparisons of nodule-related traits between two subspecies. (B, D, F) Comparisons of nodule-related traits among accessions. Bars represent the least squares mean of the data after log10 transformation, and error bars represent ±SE. Different letters on the bars indicate significant differences ($P < 0.05$) under Tukey's HSD test.

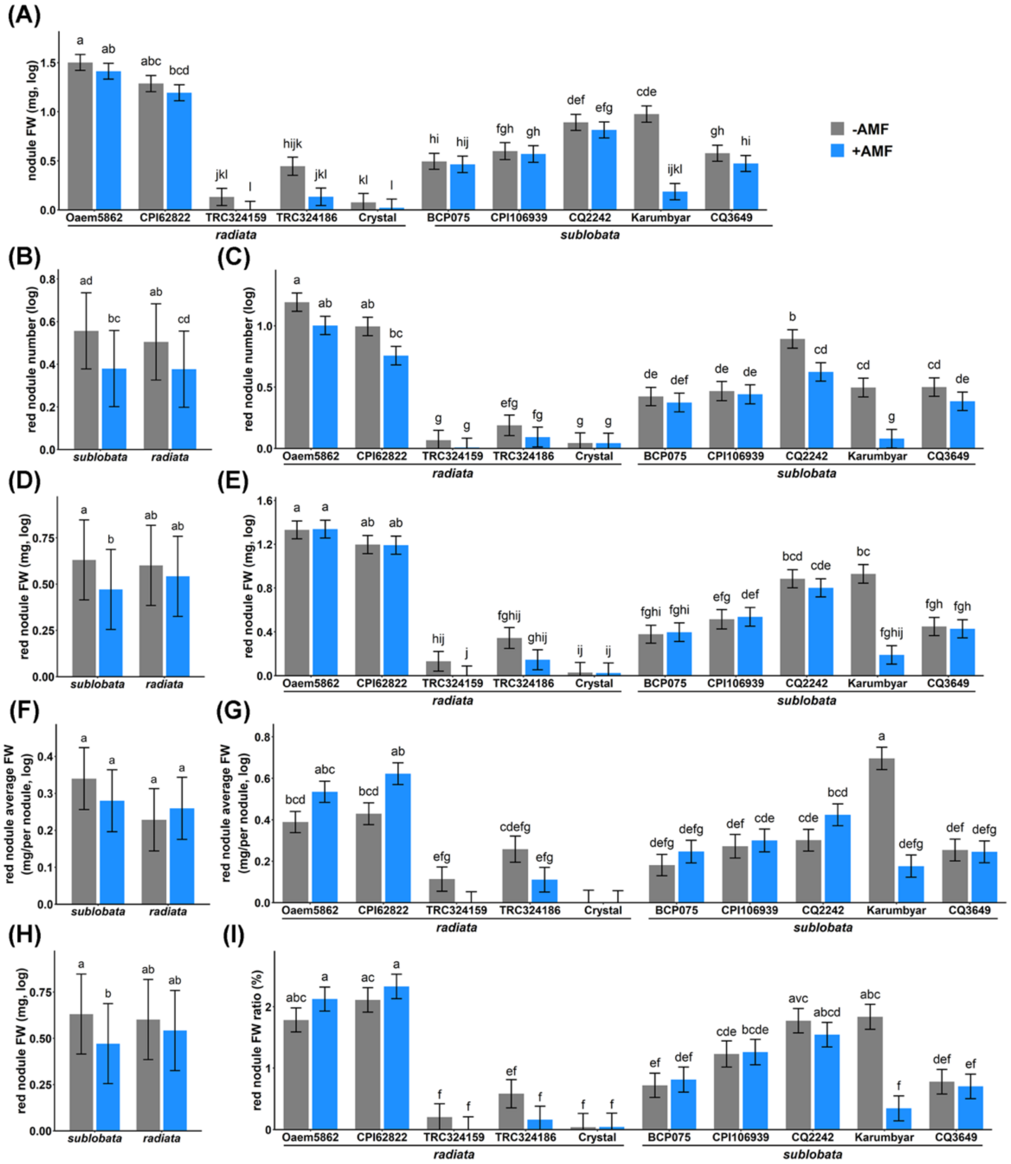

Fig. S6 Comparison of red-nodule-related traits in mungbean subspecies and accessions with *Bj* and *Bj* + *Ri* treatments. (A) Comparisons of nodule FW among different accessions. (B, D, F, H) Comparisons of red-nodule-related traits between two subspecies. (C, E, G, I) Comparisons of red nodule-related traits among different accessions. Bars represent the least squares mean of the data after log10 transformation, and error bars represent ±SE. Different letters on the bars indicate significant differences ($P < 0.05$) under Tukey's HSD test.

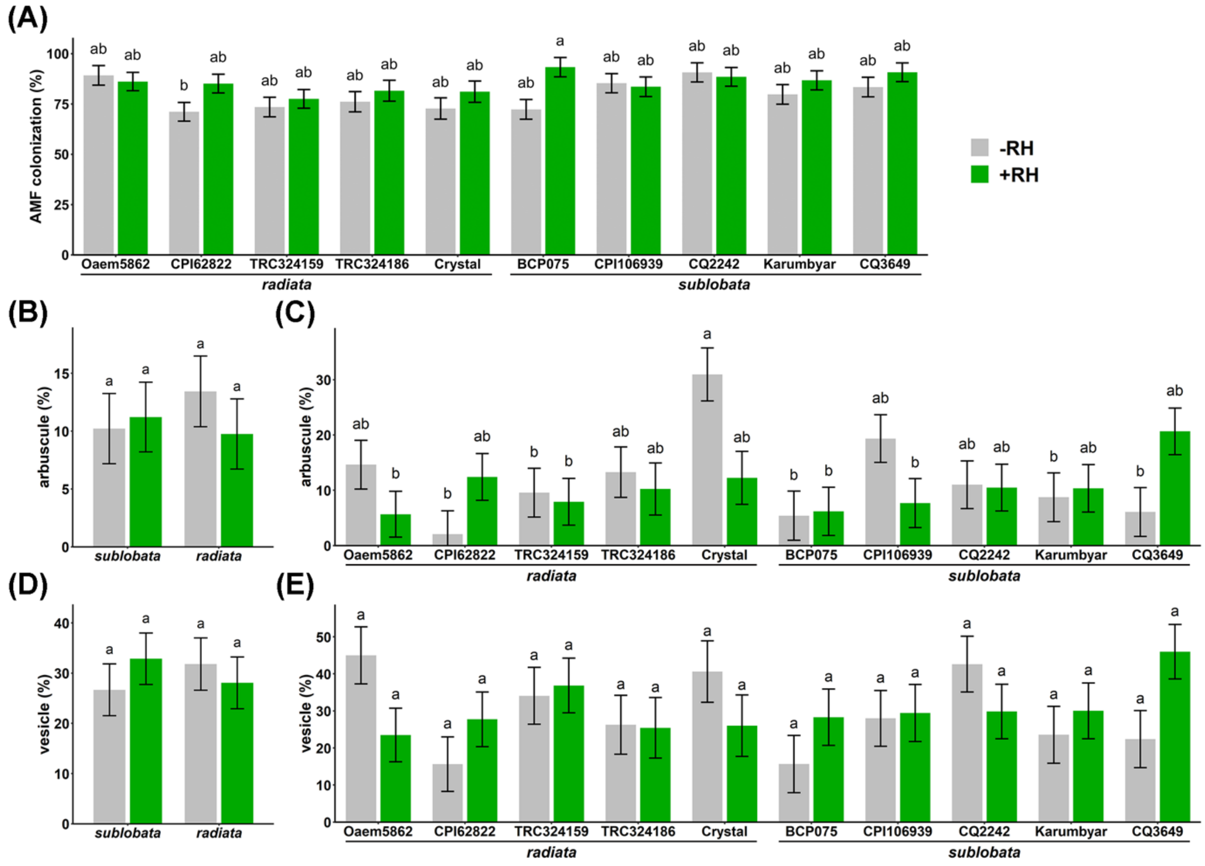

Fig. S7 Comparison of AMF-related traits in mungbean with *Ri* and *Bj* + *Ri* treatments. (A, C, E) Comparisons of AMF-related traits among different accessions. (B, D) Comparisons of AMF-related traits between two subspecies. Bars represent the least squares mean of the data after log10 transformation, and error bars represent ±SE. Different letters on the bars indicate significant differences ($P < 0.05$) under Tukey's HSD test.

Table S1. Information of 10 *V. radiata* accessions.

(A) *V. radiata* spp. *sublobata* (wild)

Accession	Country	Latitude	Longitude
BCP 075	Papua New Guinea	-6.58333	146.71666
CPI106939	Indonesia	-10	124.16666
CQ2242	Australia	-13.7	131.29999
CQ3649	Australia	-27.11666	152.54999
Karumbyar	India	11.41666	79.01666

(B) *V. radiata* spp. *radiata* (cultivar)

Accession	Code name in this study	Genetic group	Country
Oaem 5862	*NA*	East	unknown
CPI62822	*NA*	Central	unknown
Crystal	*NA*	Southeast	unknown
TRC 324159	*NA*	South	Pakistan
TRC 324186	*NA*	South	India
VI001859 BG	ACC1	Southeast	Thailand
VI002206 AG	ACC2	Southeast	Philippines
VI001339 AG	ACC3	Southeast	Philippines
VI002432 AG	ACC 4	Southeast	Thailand
VI000551 AG	ACC 5	South	India
VI001419 BG	ACC 6	South	India
VI003658 BG	ACC 7	South	India
VI004480 AG	ACC 8	Central	Iran
VI002860 AG	ACC 9	Central	Iran
VI004307 AG	ACC 10	Central	Afghanistan

VI: vegetable introduction number from the World Vegetable Center.
Others: obtained from the Australian Grains Genebank.

Table S2. The methods of trait measurement in this study.

Trait	Type [a]	Unit	Description
total FW	individual	mg	total plant fresh weight
shoot FW	individual	mg	total shoot fresh weight
leaf FW	individual	mg	leaf fresh weight
root FW	individual	mg	total root fresh weight
pure root FW	individual	mg	root FW – total nodule FW
shoot DW	individual	mg	dry the shoot part in 65℃ for 3days, and weight the dried tissue
leaf DW	individual	mg	dry the leaf part in 65℃ for 3days, and weight the dried tissue
Nunit content	pool	%	measure N content with salicylic acid method
Punit content	pool	%	measure P content with mass method
shoot Ntotal content	individual	mg	shoot N content x shoot DW
shoot Ptotal content	individual	mg	shoot P content x shoot DW
branch number	individual	branch	count the branch number of individuals
stem width	individual	mm	use vernier caliper to measure the stem width
plant height	individual	mm	measure plant height from soil to the main shoot tip with ruler
RCC	individual	no unit	use SPAD to measure
water content	individual	%	(shoot FW - shoot DW)/ shoot FW
root/shoot FW ratio	individual	%	root FW / shoot FW
pure root/shoot FW ratio	individual	%	pure root FW / shoot FW
root/total FW ratio	individual	%	root FW / total FW
unit weight	individual	mg/mm	shoot FW / height
leaf area	individual	mm^2	put 3 leaves of the same branch per plant into L-type plastic folder and scan without petiole. The leaf area was measured by image J
biggest leaf area	individual	mm^2	choose one biggest leaf per plant from the photo and measure the leaf area by image J
specific leaf area	individual	mm^2/mg	leaf area / leaf DW
total nodule number	individual	pieces	total nodule number
red nodule number	individual	pieces	red nodule number
total nodule FW	individual	mg	total nodule fresh weight per plant
red nodule FW	individual	mg	total red nodule fresh weight per plant
total nodule average FW	individual	mg/nodule	total nodule FW / total nodule number
red nodule average FW	individual	mg/nodule	red nodule FW / red nodule number
total nodule FW ratio	individual	%	red nodule weight / total plant FW
red nodule FW ratio	individual	%	red nodule weight / total plant FW
arbuscular	individual	%	observe each sight of root fragments under microscope and record the existance of the arbuscular
vesicle	individual	%	observe each sight of root fragments under microscope and record the existance of the vesicle
total AMF colonization	individual	%	observe each sight of root fragments under microscope and record the existance of the AMF structure

a. individual: each plant has one phenotypic value; pool: individuals of the same accession under the same treatment in the same batch have a phenotypic value. FW: fresh weight. DW: DW, RCC: relative chlorophyll content.

Table S3. The analysis of variance (ANOVA) of all traits after log10 transformation.

Trait	Sample size	S		Rhizobia		AMF		S*Rhizobia		S*AMF		AMF*Rhizobia		S*AMF*Rhizobia	
		F value	*P* value	*F* value	*P* value	*F* value	*P* value	*F* value	*P* value	*F* value	*P* value	*F* value	*P* value	*F* value	*P* value
total FW	709	18.06	**0.0028****	8.92	**0.0029****	19.85	**<0.0001*****	0.04	0.8489	0.22	0.6378	1.17	0.2801	1.79	0.1819
shoot FW	709	17.79	**0.0029****	6.54	**0.0108***	13.79	**0.0002*****	0.73	0.3935	0.02	0.8975	0.16	0.6886	0.58	0.4461
leaf FW	557	20.93	**0.0018****	20	**<0.0001*****	1.97	0.161	1.82	0.1774	0.37	0.5408	0.11	0.7411	0.52	0.4696
root FW	709	15.96	**0.004****	11.42	**0.0008****	23.65	**<0.0001*****	1.27	0.2609	1.02	0.3128	6.13	**0.0135***	2.81	0.094
pure root FW	709	16.44	**0.0037****	6.8	**0.0093****	22.43	**<0.0001*****	1.31	0.2527	0.93	0.3341	6.7	**0.0098****	2.93	0.0875
shoot DW	709	20.76	**0.0019****	0.32	0.5735	13.83	**0.0002*****	0.43	0.512	0.46	0.4961	0.53	0.4657	0.03	0.8717
leaf DW	707	23.92	**0.0012****	0.13	0.7235	5.78	**0.0165***	0.37	0.5446	0.01	0.9292	0.27	0.6047	0.1	0.7518
root/shoot FW ratio	709	0.62	0.4539	0.61	0.4345	3.52	0.0612	9.69	**0.0019****	3.67	0.0559	20.35	**<0.0001*****	3.24	0.0722
pure rootshoot FW ratio	709	0.63	0.4518	0.23	0.06348	3.08	0.0797	9.71	**0.0019****	3.46	0.0634	21.14	**<0.0001*****	3.39	0.0659
root/total FW ratio	709	0.25	0.6307	2.7	0.101	5.66	**0.0176***	8.85	**0.003****	2.94	0.0871	18.56	**<0.0001*****	2.55	0.111
water content	709	3.42	0.1015	42.52	**<0.0001*****	0.59	0.4414	0.02	0.8883	4.71	**0.0303***	1.31	0.2528	4.56	**0.0331***
weight per mm	709	7.79	**0.0236***	17.06	**<0.0001*****	0.06	0.8071	0.48	0.4866	0.05	0.8239	0.1	0.7503	0.02	0.8909
RCC at 4 wpi	708	0	0.9625	5.11	**0.0241***	1.95	0.1625	0.55	0.4574	3.23	0.0726	0.02	0.8928	0	0.9891
RCC at 5 wpi	709	0.06	0.8199	13.15	**0.0003*****	0.43	0.5142	0.19	0.6626	0.49	0.4841	0.7	0.403	0.08	0.7771
RCC at 6 wpi	709	0.92	0.3654	103.49	**<0.0001*****	2.04	0.1535	4.56	**0.0331***	0.54	0.4636	0.55	0.4577	0	0.9638
branch number	709	1.35	0.2796	4.52	**0.0339***	0.8	0.3722	1.94	0.1643	1.09	0.2978	2.34	0.1269	0.06	0.8041
stem width	709	19.61	**0.0022****	27.53	**<0.0001*****	11.06	**0.0009*****	0.48	0.488	0.1	0.7501	8.26	**0.0042****	2.26	0.1331
plant height	706	8.99	**0.017***	2.99	0.084	8.95	**0.0029****	0	0.9913	0.1	0.7539	0	0.9532	0.68	0.4113
leaf area	695	23.43	**0.0013****	9.35	**0.0023****	5.99	**0.0147***	1.85	0.174	0.36	0.5466	2.48	0.1158	0	0.954
biggest leaf area	709	19.72	**0.0022****	6.59	**0.0105***	2.03	0.1548	1.62	0.204	0.46	0.4969	2.39	0.1227	0.34	0.5594
specific leaf area	693	2.31	0.167	13.03	**0.0003*****	4.41	**0.0361***	8.03	**0.0047****	0.6	0.4387	1.06	0.3033	0.97	0.3252
shoot N content	671	16.98	**0.0034****	43.47	**<0.0001*****	16.34	**<0.0001*****	0.41	0.5242	8.54	**0.0036****	0.62	0.431	0.06	0.806
shoot P content	664	16.76	**0.0035****	9.52	0.0025**	4.39	**0.0365***	4.88	**0.0275***	0.23	0.6316	0.25	0.6177	0.02	0.8919
unit N content	187	1.16	0.3121	22.18	**<0.0001*****	0.05	0.8218	0.87	0.3509	2.81	0.0957	0.12	0.7258	0.73	0.5287
unit P content	185	0.19	0.6713	5.88	**0.0164***	23.54	**<0.0001*****	0.41	0.5219	1.1	0.2965	0	0.9582	0.16	0.6931

S: two mung bean subspecies in the experiment, fixed effect. Rhizobia: with or without *Bj* inoculation, fixed effect. AMF: with or without *Ri* inoculation, fixed effect. Model = S + AMF + Rhizobia+ S*AMF + S*Rhizobia + AMF*Rhizobia + S*AMF*Rhizobia + batch (random) + accession (nested within subspecies, random). FW: fresh weight, DW: dry weight, RCC: relative chlorophyll content, wpi: week after inoculation. * represents P < 0.05. ** represents P < 0.01. *** represents P < 0.001.

Table S4. The analysis of variance (ANOVA) of RCC after log10 transformation.

Effect	*F* value	*P* value
S	0.19	0.6717
Rhizobia	61.05	**<0.0001*****
AMF	0.48	0.4872
week	5.42	**0.02***
S*Rhizobia	1.75	0.1858
S*AMF	2.9	0.0887
S*week	13.72	**0.0002*****
Rhizobia*AMF	1.07	0.3001
Rhizobia*week	89.56	**<0.0001*****
AMF*week	3.71	0.0541
S*Rhizobia*AMF	0.04	0.8478
S*Rhizobia*week	4.24	**0.0397***
S*AMF*week	0.25	0.6138
S*Rhizobia*AMF*week	0	0.9498
Rhizobia*AMF*week	0.26	0.6074

S: two mung bean subspecies in the experiment, fixed effect. Rhizobia: with or without *Bj* inoculation, fixed effect. AMF: with or without *Ri* inoculation, fixed effect. Accession is a random effect nested within subspecies. Model = subspecies + AMF + Rhizobia + week + interaction among the four main effects + batch (random) + accession (nested within subspecies, random). * represents P < 0.05. ** represents P < 0.01. *** represents P < 0.001. The sample size is n=2126.

Table S5. The analysis of variance (ANOVA) of rhizobia traits after log10 transformation.

Trait	Sample size	S		AMF		S*AMF	
		F value	*P* value	*F* value	*P* value	*F* value	*P* value
red nodule number	351	0.01	0.9119	36.38	**<0.0001*** **	0.94	0.3328
total nodule number	351	0.03	0.8725	93.11	**<0.0001*** **	0.48	0.4896
red nodule FW	351	0	0.9436	10.6516	**0.0012** **	2.28	0.1321
total nodule FW	351	0	0.9615	29.28	**<0.0001*** **	1.5	0.2212
red nodule average FW	351	0.33	0.5805	0.32	0.5691	3.22	0.0736
total nodule average FW	351	0.52	0.4911	0.62	0.4326	3.08	0.0802
red nodule FW ratio	351	0.11	0.7539	3.78	0.0526	4.95	**0.0267* **
total nodule FW ratio	351	0.05	0.8281	13.37	**0.0003*** **	2.51	0.114

Data with *Bj* treatment and *Bj*+*Ri* treatment were used. S: two mung bean subspecies in the experiment, fixed effect. AMF: with or without Ri inoculation, fixed effect. Accession is a random effect nested within subspecies. Model = S + AMF + S*AMF + batch (random) + accession (nested within subspecies, random). * represents P < 0.05. ** represents P < 0.01. *** represents P < 0.001.

Table S6. The analysis of variance (ANOVA) of AMF traits after log10 transformation.

Trait	Sample size	S		Rhizobia		S*Rhizobia	
		F value	*P* value	*F* value	*P* value	*F* value	*P* value
vesicle	348	0	0.966	0.17	0.6803	2.79	0.0957
arbuscule	348	0.1	0.7639	0.55	0.4595	1.68	0.1958
AMF colonization	348	5.9	**0.0396***	9.38	**0.0024****	0.01	0.9293

Data with *Ri* treatment and *Bj*+*Ri* treatment were used. S: two mung bean subspecies in the experiment, fixed effect. Rhizobia: with or without Bj inoculation, fixed effect. Accession is a random effect nested within subspecies. Model = S + Rhizobia + S*Rhizobia + batch (random) + accession (nested within subspecies, random). * represents P < 0.05. ** represents P < 0.01. *** represents P < 0.001.

Table S7. The analysis of variance (ANOVA) of interaction among mungbean, AMF, and nodule fresh weight after log10 transformation.
(A) Analysis on all 10 mungbean accessions

Trait	Sample size	R^2	S		AMF		total nodule FW		S*AMF		S* total nodule FW		AMF* total nodule FW		S*AMF* total nodule FW	
			F value	*P* value	*F* value	*P* value	*F* value	*P* value	*F* value	*P* value	*F* value	*P* value	*F* value	*P* value	*F* value	*P* value
shoot N content	336	0.73	18.90	**0.0031****	4.53	**0.034***	45.97	**<0.0001*****	4.39	**0.0369***	7.10	**0.0082****	4.78	**0.0295***	1.24	0.2658
shoot P content	301	0.67	7.44	0.0299	5.31	**0.022***	0.01	0.9282	29.18	**<0.0001*****	9.23	**0.0026****	1.71	0.1922	0.41	0.524
total FW	351	0.77	21.75	**0.002****	0.03	0.8531	90.95	**<0.0001*****	1.82	0.1785	0.53	0.4685	4.77	**0.0296***	2.77	0.0968
shoot FW	351	0.78	17.65	**0.0034****	0.78	0.3777	91.59	**<0.0001*****	1.34	0.2483	0.03	0.855	8.07	**0.0048****	3.36	0.0675
leaf FW	274	0.68	21.32	**0.0023****	0.40	0.5292	40.36	**<0.0001*****	0.36	0.5489	1.26	0.2629	3.82	0.0516	0.00	0.9556
root FW	351	0.7	24.67	**0.0014****	0.20	0.6517	69.98	**<0.0001*****	1.93	0.1662	2.37	0.1249	1.37	0.2432	1.40	0.2377
pure root FW	351	0.69	24.82	**0.0014****	0.21	0.6493	58.11	**<0.0001*****	1.94	0.1644	2.14	0.145	1.16	0.2815	1.21	0.2728
shoot DW	351	0.78	14.35	**0.006****	2.58	0.1091	59.50	**<0.0001*****	0.49	0.4837	0.75	0.3882	7.82	**0.0055****	1.42	0.2343
leaf DW	350	0.69	21.91	**0.002****	0.56	0.4563	26.23	**<0.0001*****	0.07	0.7903	1.55	0.2148	6.64	**0.0104***	0.46	0.4988
root/shoot FW ratio	351	0.32	0.03	0.8689	3.85	0.0506	0.24	0.6216	0.08	0.7717	0.48	0.491	3.40	0.0661	0.29	0.5919
pure root/shoot FW ratio	351	0.32	0.02	0.8873	3.48	0.0629	2.07	0.1519	0.09	0.7604	0.41	0.521	3.45	0.0641	0.36	0.5489
root/total FW ratio	351	0.28	0.00	0.9541	3.44	0.0644	0.32	0.5702	0.24	0.6259	1.86	0.1735	3.08	0.0801	0.29	0.5892
water content	351	0.72	3.20	0.1137	6.26	**0.0128***	6.42	**0.0117***	1.19	0.277	10.13	**0.0016****	1.35	0.2453	2.13	0.1455
weight per mm	351	0.38	6.08	**0.043***	0.17	0.6832	9.99	**0.0019****	0.21	0.6478	2.34	0.1275	0.00	0.967	0.17	0.6798
RCC at 4 wpi	351	0.44	0.00	0.9985	0.40	0.5298	1.53	0.2163	1.69	0.1951	0.14	0.7115	0.05	0.8316	1.33	0.25
RCC at 5 wpi	351	0.44	0.01	0.9296	0.70	0.405	0.77	0.3816	1.33	0.2496	0.08	0.7754	4.10	**0.0436***	0.28	0.5997
RCC at 6 wpi	351	0.42	0.10	0.7626	0.34	0.5598	22.01	**<0.0001*****	0.37	0.5424	0.09	0.7608	3.31	0.0698	0.97	0.3245
branch number	351	0.6	1.26	0.296	0.54	0.4646	10.53	**0.0013****	0.96	0.3268	0.59	0.443	0.73	0.3921	0.25	0.6164
stem width	351	0.69	13.99	**0.0069****	10.01	**0.0017****	31.39	**<0.0001*****	1.16	0.2823	3.76	0.0533	1.97	0.1613	1.10	0.2946
plant height	351	0.48	10.18	**0.0127***	1.36	0.244	12.11	**0.0006*****	1.89	0.1699	4.96	**0.0272***	4.39	**0.0369***	3.43	0.065
leaf area	344	0.72	25.03	**0.0012****	1.06	0.3045	43.73	**<0.0001*****	0.14	0.707	2.79	0.0964	1.89	0.1699	0.05	0.8222
biggest leaf area	351	0.73	18.97	**0.0028****	0.06	0.8029	44.46	**<0.0001*****	0.36	0.5502	2.99	0.0851	3.74	0.054	0.18	0.6687
specific leaf area	343	0.79	3.63	0.0962	0.76	0.3855	0.07	0.7955	0.36	0.5504	0.99	0.3213	2.58	0.1092	0.06	0.8039

(Continue)

(B) Analysis on *V. radiata* spp. *sublobata* only

Trait	Sample size	R^2	AMF		total nodule FW		AMF* total nodule FW	
			F value	*P* value	*F* value	*P* value	*F* value	*P* value
shoot N content	176	0.58	0.51	0.477	21.27	**<.0001*** **	7.16	**0.0082** **
shoot P content	149	0.56	14.39	**0.0002*** **	22.14	**<.0001*** **	8.85	**0.0034** **
total FW	183	0.57	1.36	0.2454	63.87	**<.0001*** **	7.04	**0.0087** **
shoot FW	183	0.61	0.35	0.555	58.31	**<.0001*** **	9.70	**0.0022** **
leaf FW	144	0.48	1.09	0.2983	29.91	**<.0001*** **	1.37	0.2436
root FW	183	0.48	2.10	0.149	50.87	**<.0001*** **	2.61	0.108
pure root FW	183	0.45	2.08	0.151	42.45	**<.0001*** **	2.29	0.1324
shoot DW	183	0.58	0.06	0.8056	34.15	**<.0001*** **	7.97	**0.0053** **
leaf DW	183	0.47	0.01	0.9204	26.25	**<.0001*** **	4.75	**0.0307***
root/shoot FW ratio	183	0.39	1.51	0.2213	0.01	0.9111	1.79	0.1821
pure root/shoot FW ratio	183	0.4	1.37	0.2433	0.54	0.4617	1.88	0.1726
root/total FW ratio	183	0.36	1.66	0.1997	0.93	0.3356	1.57	0.2114
water content	183	0.49	6.13	**0.0143***	14.13	**0.0002*** **	0.01	0.9179
weight per mm	183	0.46	0.15	0.7015	8.45	**0.0041** **	0.33	0.5678
RCC at 4 wpi	183	0.58	0.00	0.9561	0.24	0.6218	0.00	0.9683
RCC at 5 wpi	183	0.52	0.28	0.5998	4.92	**0.0277***	3.33	0.0697
RCC at 6 wpi	183	0.42	0.12	0.7252	14.76	**0.0002*** **	2.56	0.1117
branch number	183	0.19	1.12	0.291	7.13	**0.0085** **	0.09	0.7669
stem width	183	0.35	1.91	0.169	11.01	**0.0011** **	3.61	0.059
plant height	183	0.41	0.08	0.7732	17.16	**<.0001*** **	5.03	**0.0262***
leaf area	180	0.58	0.02	0.8812	33.87	**<.0001*** **	1.49	0.2243
biggest leaf area	183	0.59	0.39	0.5355	40.95	**<.0001*** **	3.13	0.0788
specific leaf area	180	0.69	0.36	0.5469	0.17	0.6829	0.87	0.3529

(Continue)

(C) Analysis on *V. radiata* spp. *radiata* only

Trait	Sample size	R^2	AMF		total nodule FW		AMF* total nodule FW	
			F value	*P* value	*F* value	*P* value	*F* value	*P* value
shoot N content	160	0.71	8.61	**0.0039****	26.87	**<.0001*****	1.08	0.3008
shoot P content	152	0.42	1.54	0.2172	17.88	**<.0001*****	0.47	0.4937
total FW	168	0.57	0.94	0.3328	33.86	**<.0001*****	0.18	0.6742
shoot FW	168	0.61	2.23	0.1372	37.7	**<.0001*****	1.05	0.3079
leaf FW	168	0.45	0.02	0.8823	10.65	**0.0023****	4.77	**0.0309***
root FW	168	0.47	0.19	0.6608	25	**<.0001*****	0.04	0.8496
pure root FW	168	0.45	0.20	0.6552	20.87	**<.0001*****	0.04	0.8434
shoot DW	168	0.6	3.04	0.0832	28.71	**<.0001*****	3.09	0.0807
leaf DW	168	0.47	1.02	0.314	5.64	**0.0197***	5.25	**0.0233***
root/shoot FW ratio	168	0.31	3.23	0.0741	0.3	0.5846	3.6	0.0595
pure root/shoot FW ratio	168	0.31	2.87	0.092	1.75	0.1917	3.35	0.0691
root/total FW ratio	168	0.27	2.21	0.1388	0.07	0.797	3.19	0.076
water content	168	0.76	1.56	0.2135	0.02	0.8857	9.65	**0.0023****
weight per mm	168	0.29	0.11	0.7431	4.97	**0.0344***	0.08	0.7805
RCC at 4 wpi	168	0.38	1.15	0.285	3.02	0.0916	2.12	0.1473
RCC at 5 wpi	168	0.44	0.15	0.6997	0.07	0.7921	3.6	0.0595
RCC at 6 wpi	168	0.46	0.06	0.8112	8.26	**0.0054****	1.21	0.2733
branch number	168	0.71	0.01	0.9423	4.44	**0.0368***	2.25	0.1356
stem width	168	0.52	7.12	**0.0084****	21.43	**<.0001*****	0.09	0.7636
plant height	168	0.4	2.08	0.1512	1.42	0.24	0.14	0.7106
leaf area	164	0.7	1.61	0.2058	11.43	**0.0014****	1.96	0.1639
biggest leaf area	168	0.72	0.66	0.4171	10.28	**0.0019****	3.25	0.0735
specific leaf area	163	0.87	0.23	0.6308	0.59	0.4446	4.84	**0.0294***

Data with *Bj* treatment and *Bj*+*Ri* treatment were used. S: two mung bean subspecies in the experiment, fixed effect. AMF: with or without Ri inoculation, fixed effect. Accession is a random effect nested within subspecies. Model = S + AMF + total nodule weight + S*AMF + S* total nodule weight + AMF* total nodule weight + S*AMF* total nodule weight + batch (random) + accession (nested within subspecies, random). * represents P < 0.05. ** represents P < 0.01. *** represents P < 0.001.

Table S8. The heritability analysis of all traits was measured after log10 transformation in this study.

(A) Plant traits

	Heritability					*Qst*				
Trait/Treatment	control	AMF	Rhizobia	AMF+Rhizobia	Mean	control	AMF	Rhizobia	AMF+Rhizobia	Mean
unit N content	0.02	0.10	0.20	0.26	0.15	0.00	0.31	0.00	0.33	0.16
unit P content	0.20	0.36	0.29	0.25	0.28	0.00	0.00	0.00	0.00	0.00
shoot N content	0.58	0.40	0.58	0.37	0.48	0.82	0.69	0.66	0.75	0.73
shoot P content	0.72	0.72	0.76	0.62	0.71	0.78	0.68	0.76	0.83	0.76
RCC at 4 wpi	0.18	0.29	0.12	0.14	0.18	0.00	0.00	0.00	0.00	0.00
RCC at 5 wpi	0.27	0.29	0.22	0.18	0.24	0.00	0.00	0.00	0.00	0.00
RCC at 6 wpi	0.32	0.37	0.26	0.26	0.30	0.00	0.03	0.00	0.00	0.01
branch number	0.59	0.58	0.72	0.51	0.60	0.19	0.09	0.00	0.00	0.07
stem width	0.74	0.76	0.76	0.67	0.73	0.76	0.82	0.83	0.78	0.80
plant height	0.39	0.43	0.44	0.32	0.40	0.67	0.60	0.66	0.61	0.64
total FW	0.76	0.68	0.80	0.72	0.74	0.75	0.76	0.80	0.81	0.78
shoot FW	0.76	0.74	0.81	0.71	0.76	0.81	0.76	0.75	0.79	0.78
leaf FW	0.65	0.69	0.69	0.66	0.67	0.89	0.81	0.74	0.80	0.81
root FW	0.72	0.54	0.74	0.66	0.67	0.62	0.72	0.83	0.82	0.75
pure root FW	0.72	0.54	0.74	0.67	0.67	0.62	0.72	0.84	0.83	0.75
root/shoot FW ratio	0.49	0.12	0.29	0.24	0.29	0.22	0.00	0.00	0.00	0.06
root/total FW ratio	0.47	0.10	0.26	0.20	0.26	0.18	0.00	0.00	0.00	0.05
water content	0.77	0.47	0.70	0.70	0.66	0.46	0.14	0.34	0.26	0.30
shoot DW	0.77	0.77	0.81	0.73	0.77	0.86	0.78	0.78	0.80	0.81
leaf DW	0.68	0.68	0.70	0.66	0.68	0.86	0.80	0.79	0.85	0.83
unit weight	0.40	0.41	0.35	0.36	0.38	0.59	0.68	0.48	0.67	0.61
leaf area	0.55	0.57	0.49	0.43	0.51	0.86	0.83	0.73	0.84	0.82
biggest leaf area	0.50	0.54	0.47	0.41	0.48	0.83	0.80	0.74	0.79	0.79
specific leaf area	0.15	0.14	0.17	0.17	0.16	0.15	0.00	0.40	0.34	0.22

(B) Rhizobia traits

	Heritability					*Qst*				
Trait/Treatment	control	AMF	Rhizobia	AMF+ Rhizobia	Mean	control	AMF	Rhizobia	AMF+ Rhizobia	Mean
red nodule number	-	-	0.69	0.62	0.66	-	-	0	0	0.00
total nodule number	-	-	0.77	0.64	0.71	-	-	0	0	0.00
red nodule FW	-	-	0.66	0.68	0.67	-	-	0	0	0.00
total nodule FW	-	-	0.74	0.7	0.72	-	-	0	0	0.00
red nodule average FW	-	-	0.43	0.44	0.44	-	-	0	0	0.00
total nodule average FW	-	-	0.43	0.45	0.44	-	-	0	0	0.00
red nodule ratio	-	-	0.44	0.59	0.52	-	-	0	0	0.00
total nodule ratio	-	-	0.56	0.62	0.59	-	-	0	0	0.00

(C) AMF traits

	Heritability					*Qst*				
Trait/Treatment	control	AMF	Rhizobia	AMF+Rhizobia	Mean	control	AMF	Rhizobia	AMF+Rhizobia	Mean
vesicle	-	0.09	-	0.02	0.06	-	0	-	0.35	0.18
arbuscule	-	0.12	-	0.03	0.08	-	0	-	0	0.00
AMF colonization	-	0.06	-	0.08	0.07	-	0.15	-	0.92	0.54

Heritability: estimates proportion of genetic variation, including subspecies effect and accession effect in the full model.
Qst: estimates proportion of subspecies genetic variation in heritability.

Table S9. The analysis of variance (ANOVA) of 20 mungbean accessions inoculated with two rhizobia after log10 transformation.

Trait	Sample size	group		treatment		group*treatment	
		F value	*P* value	*F* value	*P* value	*F* value	*P* value
RCC at 4wpi	146	0.96	0.4359	0.51	0.4781	0.54	0.6566
RCC at 5wpi	146	0.76	0.5367	15.77	**0.0001*** **	2.08	0.1069
RCC at 6wpi	146	0.39	0.7598	31.72	**<0.0001*** **	3.15	**0.0274***
total FW	146	23.33	**<0.0001*** **	0.23	0.6332	1.94	0.1274
shoot FW	146	20.60	**<0.0001*** **	2.02	0.1579	1.32	0.2718
root FW	146	22.24	**<0.0001*** **	4.02	**0.0473***	2.21	0.0908
pure root FW	146	21.68	**<0.0001*** **	5.45	**0.0212***	2.22	0.0890
total DW	146	23.83	**<0.0001*** **	0.01	0.9080	2.84	**0.0405***
shoot DW	146	20.68	**<0.0001*** **	0.64	0.4266	1.75	0.1611
root DW	146	24.02	**<0.0001*** **	2.24	0.1372	5.02	**0.0026*** *
pure root DW	146	20.76	**<0.0001*** **	2.29	0.1324	4.71	**0.0038*** *
water content (%)	146	4.40	**0.0194***	2.89	0.0916	1.09	0.3541
root/shoot FW ratio (%)	146	3.34	**0.0476***	21.58	**<0.0001*** **	1.36	0.2585
root/total FW ratio (%)	146	3.11	0.0573	20.85	**<0.0001*** **	1.19	0.3158
root/shoot DW ratio (%)	146	4.05	**0.0282***	7.65	**0.0066*** *	2.36	0.0745
root/total DW ratio (%)	146	4.90	**0.0149***	8.04	**0.0054*** *	2.12	0.1015
total nodule number	146	1.40	0.2786	111.30	**<0.0001*** **	6.46	**0.0004*** **
total nodule FW	146	2.51	0.0969	48.47	**<0.0001*** **	8.12	**0.0001*** **
total nodule average FW	146	1.93	0.1710	1.17	0.2823	10.38	**<0.0001*** **
total nodule DW	146	3.90	**0.0302***	17.41	**0.0001*** **	2.58	0.0568
total nodule average DW	146	1.57	0.2403	0.01	0.9175	3.52	**0.0173***
total nodule FW ratio	146	1.31	0.3075	50.21	**<0.0001*** **	2.20	0.0910
total nodule DW ratio	146	0.71	0.5631	0.75	0.3890	0.60	0.6136

group: three groups of cultivars and one wild mungbean group in the experiment, fixed effect. treatment: inoculated with *Bj* or *Sf*. Accession is a random effect nested within subspecies. Model = group + treatment + group*treatment + accession (nested within subspecies, random). * represents P < 0.05. ** represents P < 0.01. *** represents P < 0.001.
